# Help-seeking behaviors among survivors of intimate partner violence during pregnancy in 54 low- and middle-income countries: evidence from Demographic and Health Survey data

**DOI:** 10.1186/s12889-025-21421-3

**Published:** 2025-02-01

**Authors:** Mariella Stiller, Michael Lowery Wilson, Till Bärnighausen, Adebola Adedimeji, Erin Lewis, Anne Abio

**Affiliations:** 1 Heidelberg Institute of Global Health (HIGH), University of Heidelberg, Heidelberg, Germany; 2 Injury Epidemiology and Prevention Research Group, University of Turku, Turku, Finland; 3 Departments of Social Sciences and Health Policy and Implementation Science, Wake Forest University School of Medicine, Winston Salem, NC USA; 4 Department of Epidemiology and Population Health, Albert Einstein College of Medicine, New York, USA; 5 Jacobi Medical Center, New York, USA; 6 Research Centre for Child Psychiatry, University of Turku, Turku, Finland; 7 Invest Research Flagship, University of Turku, Turku, Finland

**Keywords:** Intimate partner violence, Help-seeking, Pregnancy, Low- and middle-income countries, Demographic and Health Surveys, Maternal health

## Abstract

**Background:**

Intimate partner violence (IPV) during pregnancy is a global issue of public health importance. Due to the low rates of help-seeking in response to IPV, research on survivors’ help-seeking behaviors is scarce, particularly within low- and middle-income countries (LMICs). The present study provides a cross-national evidence of informal and formal help-seeking patterns among pregnant women experiencing IPV.

**Methods:**

This study made use of population-based data from the Demographic and Health Surveys (DHS) Program from 54 LMICs, collected between 2005 and 2020 (N = 359,027). Applying bivariate and multivariable analyses, the present study examined IPV survivors’ help-seeking distributions and associations with individual, partner, family, and community factors.

**Results:**

Only half of survivors sought help in response to IPV during pregnancy with wide regional and national variations, mainly from informal support networks (including family, neighbors, and friends), and rarely from formal institutions (including legal, socio-cultural, and medical services). Evidence shows that help-seeking behaviors were associated with IPV survivors’ age and educational attainment, survivors’ employment status and earnings compared to their partners, survivors’ consumption of mass media, intimate partner’s age and education, spouse’s alcohol consumption and controlling behaviors, survivors’ wealth index, place of residence, and health-seeking barriers, among others.

**Conclusion:**

Practitioners are encouraged to consider the study’s outcomes when designing interventions and support for survivors seeking help in response to IPV during pregnancy. Strong advocacy and action are needed, including fostering survivors’ educational attainment, diminishing pregnant women’s social and financial dependencies on their intimate partners, promoting pre- and peri-natal health care, informing survivors about help, and increasing gender equality by engaging women and men equally within the whole community.

## Introduction

Intimate partner violence (IPV) is a serious global public health challenge, a major human rights violation, and the most common form of violence against women worldwide [1]. However, IPV is rarely disclosed, and survivors’ help-seeking behaviors remain poorly studied, particularly within low- and middle-income countries (LMICs) and during the vulnerable period of pregnancy [2–5].

Pregnancy can be a particularly delicate and stressful period, as it can entail higher stress levels due to rising physical, psychological, emotional, social, and economic burdens, which in turn can imply a higher vulnerability for pregnant women to be abused in an intimate partnership [4, 6–9], which substantiates the research focus on this specific population at risk.

IPV is defined as any act of physical aggression, psychological abuse, sexual coercion or controlling behaviours by a current or former partner or spouse [1]. Prevalence rates for IPV during pregnancy vary widely between studies and between regions. In a cross-country study analysing data of IPV during pregnancy from 19 countries between 1998 and 2007, more than half of the surveys revealed a lifetime prevalence ranging between 3.9 and 8.7%, with higher rates in Latin America and Africa compared to Europe and Asia [10]. Consistently, in other studies using data between 2000 and 2010 the highest rates for IPV during pregnancy were also reported in LMICs, but much higher prevalence rates were disclosed with 53.3% in Latin America [11] and 57% in sub-Saharan Africa [12]. Additionally, previous studies indicate that violence experienced by women tends to be exacerbated during crises, such as forced displacement, conflict, and epidemics [13, 14]. Data emerging from the SARS-CoV-2/COVID-19 pandemic has shed new light on IPV against women under such situations. Given “stay-at-home” public health mandates - and the associated decrease in access to support and medical services, in addition to socio-economic impacts - evidence has emerged indicating an increased frequency of IPV in this context due to social isolation and prolonged exposure to abusive partners [14].

Violence against pregnant women has numerous deleterious health outcomes for women, children, and families, including physical, mental, sexual, and reproductive health problems, as well as negative socio-economic consequences for countries and societies; outcomes, ranging from miscarriage, stillbirth, preterm birth, increased child mortality and morbidity, and sexually transmitted infections to maternal death, homicide and suicide have been well-described in the literature [15–20]. Seeking help when experiencing violence can be a challenging and complex process and might be even more intricate during the vulnerable period of pregnancy. Help-seeking has been defined as “violence disclosure in order to obtain a form of assistance” [5] and can be formal/institutional (e.g., police, medical professionals, legal or social service actors), or informal/non-institutional (e.g., family and friends). Liang et al. [3] conceptualized a theoretical framework for the process of help-seeking which includes three stages; (1) IPV-exposed women must recognize and define violence as a problem, (2) they must decide whether to seek help, and finally (3) they must select a help provider; all process stages are themselves influenced by individual, interpersonal and sociocultural factors. A complementary framework is the *Theory of Help-Seeking Behavior* which explains how help-seeking is influenced by survivor’s individual agency, beliefs, and social context [21]; (1) individual agency entails the self-efficacy, inner strength and intentionality to seek help; (2) beliefs include current and community experiences, as well as historical knowledge about help providers; (3) social context comprises the community influence, positionality, racial discrimination and systemic racism which also have impact on survivors’ help-seeking behaviors. As pregnancy itself can be a phase of extreme change, adaptations, and various interdependencies, these individual and sociocultural beliefs might start to falter particularly during the period of pregnancy and therefore might challenge the process of help-seeking, which this study aims to explore further.

Research has demonstrated that seeking social support - both from informal and formal sources - improves IPV survivors’ coping mechanisms, protecting them against ongoing violence and negative physical and mental health outcomes, including anxiety, depression, post-traumatic stress disorder, and suicide attempts [3, 22]. Social support might be beneficial for IPV survivors by fostering their psychological well-being in general, giving assistance in defending one’s integrity and safety, helping to imagine alternatives to the current violent situation, providing emotional, physical, financial, or material help, and encouraging to seek any further help in order to stop IPV [3, 22]. Moreover, health care providers’ screening and referral to supportive counselling for IPV survivors has been shown to improve quality of life, family cohesiveness, and increase the use of safety behaviors and community resources [23]. Although antenatal care and other health care providers can be often the first and most frequent support service for pregnant women experiencing IPV, research has also shown that IPV survivors in LMICs are less likely to use antenatal care [24], which reinforces the investigation of help-seeking behaviors within and especially outside the health care systems in LMICs.

Despite the high prevalence of IPV among women, rates of help-seeking and reporting of violence are relatively low worldwide, especially in resource-poor settings. Cross-national studies in LMICs revealed help-seeking rates between 35 and 40% among women experiencing physical and/or sexual violence; estimates range from 22.5% in Southeast Asia to 72.2% in Latin America [11, 25–27]. Most IPV survivors - with a global range from 17.6% in sub-Saharan Africa to 65.5% in Central America - first seek help from informal sources, beginning with close relatives, followed by in-laws, and friends [11, 26, 27]. By contrast, only few women seek help from formal institutions, such as police, social service organizations, and medical professionals; formal help-seeking rates vary regionally from 2% in India and East Asia to 36% in Latin America [11, 26, 27]. A study conducted in Norway reported that, compared to non-pregnant women, a significantly lower number of pregnant women disclosed their experiences with physical or psychological IPV to care providers [28], which is why a particular attention should be paid to this vulnerable group.

Seeking help for IPV is shown to be associated with multitudinous facilitating and inhibiting factors. Existing literature on this topic tends to be country-specific and focusing primarily on non-pregnant women. These studies revealed that women’s employment, education, age, marital status, and the severity of violence were associated with help-seeking [5, 26, 27, 29, 30]. In addition, women’s perception of violence as “not serious enough” or “normal” - in addition to being a source of shame and embarrassment, as well as evoking fear of disclosure leading to concerns about privacy and confidentiality - is associated with decreased support-seeking efforts [5, 11, 31, 32]. In the converse, having an employed partner, partner’s older age, and lower alcohol consumption - as well as having minor children in the home - positively influence battered women’s help-seeking [5, 27, 29–33]. Community and societal factors also play an essential role in IPV survivors’ decision to seek help, and the impact of cultural and religious beliefs, urban versus rural residence, and wealth status have also been areas of research in societies with known gender inequities [5, 7, 26, 30, 32, 34].

Economic and marital dependency theories claim that women with lower financial resources might be more dependent on their male partners and therefore less able to leave an abusive partner or to seek help [6, 7, 35], and these dependencies can aggravate particularly during pregnancy.

Whereas research attention has been devoted to the prevalence, risk factors and health outcomes associated with IPV among pregnant women in LMICs [4, 12, 20, 36], there remains a paucity of literature investigating factors associated with help-seeking in these settings. The relatively high IPV rates during pregnancy and simultaneously the relatively low rates of help-seeking in LMICs compared to high-income countries, as presented above, substantiate the study’s special focus on these economically disadvantaged environments. Moreover, there is a scarcity of population-based research examining the help-seeking behaviors of IPV survivors which is mostly country-specific [29, 30, 33, 37, 38]; only two studies - to date - have compared reporting patterns on a global scale [26, 27]. In addition, while previous cross-sectional help-seeking studies examined non-pregnant women [5, 11, 26, 27, 33], none focused only on the IPV experience of pregnant women, in spite of evidence that the latter are particularly vulnerable to violence [4, 28, 39, 40]. Finally, prior research has put a focus on formal health seeking behaviors [23, 24, 37, 41, 42] leading to a need for studies differentiating between formal and informal help-seeking behaviors.

To bridge this essential research gap, the present study aims to: 1) assess the degree to which women experiencing IPV during pregnancy sought help; 2) examine the sources of informal and formal support for this population; and 3) evaluate factors associated with help-seeking among this sample by investigating individual, family, and community-level characteristics. Leveraging this reliable and accurate data source to provide novel perspectives into help-seeking among survivors of violence during pregnancy is crucial to tailor policies, interventions, and networks to improve cross-national infrastructure to support IPV survivors during pregnancy and beyond. In sum, this study is the first research - to our knowledge - which examines formal and informal help-seeking behaviors on a global scale while putting an emphasis on IPV during pregnancy in LMICs.

## Methods

### Data

This study utilized extant, publicly available data collected by the Demographic and Health Surveys (DHS) Program from 54 LMICs. The DHS Program provides cross-sectional, population-based, and nationally representative data and collects information about health and population trends from over 90 developing countries, enabling comparisons across different nations. Within the DHS sample, only one woman per household was randomly selected to participate in the Domestic Violence module in which she was interviewed about violence perpetrated by her current or most recent husband or partner.

The present study focused on data collected between 2005 and 2020 (the fifth to eight survey waves) from currently or previously married and cohabiting females of reproductive age (15-49 years) living within the selected 54 LMICs (N = 359,027); the study focused on respondents living in the following four regions: Asia and Melanesia (12 countries), Latin America and Caribbean (6 countries), North Africa/West Asia/Eastern Europe (6 countries), and sub-Saharan Africa (30 countries) [43]. The classification into LMICs is based on the World Bank Atlas method, which defines low-income countries as those with a gross national income per capita of 1,085 U.S. Dollars or less, and middle-income countries as those with a gross national income per capita between 1,086 and 13,205 U.S. Dollars per year [44]. The term “global/globally” is used throughout the text and figures to refer to the specific set of 54 LMICs and does not represent the global distribution of all countries worldwide.

Intimate partner violence is a very personal and sensitive topic, therefore the DHS Program applied strict ethical and safety procedures which correspond to international recommendations for research on domestic violence against women [45]. Interviews were only conducted after a respondent provided informed consent and her privacy was insured; all methods were performed in accordance with the relevant regulations and guidelines. The datasets used for the present study are publicly available in anonymized form (www.dhsprogram.com/data/ available-datasets.cfm), and additional information about the DHS Program, surveys, procedures and methodology can be obtained from the official website (www.dhsprogram.com).

### Measurements

Within the Domestic Violence module, women and girls aged 15-49 years who have ever been pregnant where asked “Has anyone ever hit, slapped, kicked, or done anything else to hurt you physically while you were pregnant?” with a yes-or-no-answer. All women who answered the following question “Who has done any of these things to physically hurt you while you were pregnant?” with “current husband/partner”, “former husband/partner”, “current boyfriend”, or “former boyfriend” were coded as denominators in the present study. The dependent variable was a dichotomous question of whether a woman sought help, conditional on having experienced IPV: “Thinking about what you yourself have experienced among the different things we have been talking about, have you ever tried to seek help?”. If the respondent answered “yes” to this screening question, she was then asked from whom she sought help. Women who reported seeking help from her own family, her husband/partner’s family, her friend, or her neighbor were coded as seeking “informal” or “non-institutional” help. On the other hand, women were classified as seeking “formal” or “institutional” help if they sought help from any of the following sources: police, doctor/medical professional, religious leader, lawyer, or social service organization. Women could select multiple help-seeking sources, hence these formal and informal help categories are not mutually exclusive. Possible answer options varied by country survey.

Socio-demographic background characteristics - measured in association with women’s help-seeking - were derived from the Woman’s and the Household Questionnaires. These results were analyzed and interpreted to ascertain the facilitating and inhibiting factors for help-seeking among women who have experienced IPV during pregnancy. Independent variables were classified into three main categories: (1) women’s individual characteristics, including age at the time of survey, age at first cohabitation, marital status, highest level of education, employment status at the time of survey, detail concerning the highest wage earner in the home, exposure to mass media at least once a week (including reading newspaper or magazine, listening to radio, and watching television), and whether the current pregnancy was planned; (2) partner/family characteristics, namely partner’s age, highest level of education, employment status at the time of respondent participation in the study, partner’s alcohol consumption, number of living children, whether the respondent witnessed her father “beat” her mother, and whether a respondent is afraid of her partner; and (3) community/societal characteristics included place of residence (urban/rural), wealth index, respondent’s freedom in decision-making, perceived controlling behaviors, attitudes towards wife beating, and barriers to access health care. For the purpose of the present study, the decision-making variable included the four targeted questions concerning the respondent’s final say in the following decisions: (a) health care (b) large household purchases (c) visits to family or friends, and (d) uses for spouses’ income. For the decision-making variable, we only included the possible answers “woman alone”, “woman and partner”, and “partner alone”, and excluded the answers “woman and other person”, “someone else”, “other” and “relatives” due to the focus on intimate partners, as well as due to survey inconsistencies and alignment with other studies.

Additionally, a question concerning perceived primary barriers to help-seeking was included in surveys administered in the Dominican Republic, Peru, Burkina Faso, and Ivory Coast. A hypothesis-driven approach was applied for the selection of the independent variables of interest for this study. Based on previous research concerning factors significantly facilitating or inhibiting help-seeking [26, 27, 29, 30, 33, 37, 38], the aforementioned variables were selected for inclusion in the bivariate and multivariable analyses; the selection process was limited by inconsistencies in variable wording and answer options, as well as partial unavailability of data.

### Statistical analyses

RStudio (Version 1.4.1717) [46] was used to conduct all statistical analyses and Microsoft PowerPoint (Version 2203) was used to generate the study figures/maps. The survey sample weights for the domestic violence module were applied as recommended by the DHS to adjust for non-response disparities and differences in sampling design. The weights were divided by 1,000,000 (code in R program: dta$wt <- dta$d005/1000000) and a complex sample design was created (code in R program: DHSdesign <- svydesign(id=dta$v021, strata=dta$v023, weights=dta$wt, data=dta). The weighted data can be considered representative for a female individual - aged between 15 and 49 years - for any given nation included in the study. More information about the application of weights can be obtained from the official Guide to DHS Statistics which is available on the official DHS website: https://www.dhsprogram.com/pubs/pdf/DHSG1/Guide_to_DHS_Statistics_DHS-7.pdf.

First, the prevalence of help-seeking behaviors among survivors of IPV during pregnancy was estimated across the 54 nations by country, region, and then globally. Additionally, the proportions of IPV during pregnancy were analyzed. Secondly, bivariate analyses were performed to examine the socio-demographic background characteristics among IPV survivors who sought help. In the latter, Pearson’s Chi-square test for categorical variables and Welch’s t-test for the continuous variable (number of children) were used to test for significance.

Additionally, multivariable logistic regression models were conducted to explore the associations between women’s help-seeking behaviors and their individual, partner/family, and community/societal characteristics. In detail, three different regression models were applied to examine IPV survivors’ inhibiting and facilitating factors for: seeking any form of help (Model 1), seeking help from informal support networks, such as family, in-laws, neighbors, and friends (Model 2), and seeking help from formal sources including police, medical professionals, lawyers, religious leaders, and social service organizations (Model 3).

For bivariate and multivariable analyses, data was pooled by region (Asia and Melanesia, Latin America and Caribbean, North Africa/West Asia/Eastern Europe, sub-Saharan Africa, and Total). Because only unweighted data was available for Peru, data for this country was excluded from regional and total weighted estimates.

The models present the adjusted Odds Ratios (aORs) - and their corresponding 95% confidence intervals (CI) - to quantify the magnitude of measures of association. ORs were adjusted for potential confounders, including respondent’s age at the time of survey, age at first cohabitation, highest level of education, working status, marital status, number of living children, partner’s highest level of education and working status, whether respondent’s partner consumed alcohol, whether respondent’s father abused her mother, if respondents were afraid of their intimate partner, local wealth index, and whether respondents resided in a rural or urban locale. For both preliminary and confirmatory analyses, statistical significance was considered at a p-value of <0.05.

## Results

**Prevalence of intimate partner violence during pregnancy**

**Fig. 1:**
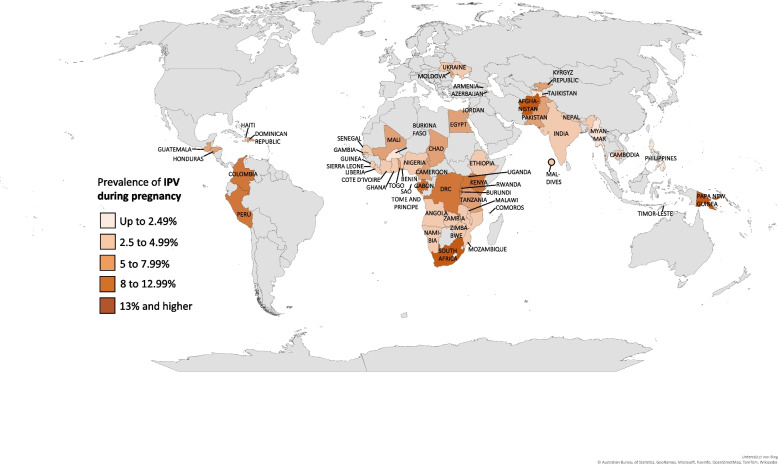
Global map of intimate partner violence during pregnancy Notes: Global map of the prevalence distribution of IPV during pregnancy among women aged 15-49 years, DSH 2005 – 2020. Results are divided into the adjacent five different colour ranges. Results are derived from Table 1

Table 1 (Prevalence of IPV during pregnancy and help-seeking behavior by country, weighted) provides the prevalence of IPV during pregnancy and help-seeking behavior, disaggregated by country and region. Overall - among a pooled global sample of 359,027 women - the prevalence of IPV during pregnancy was 5.63%; the highest regional estimate of 7.34% was identified in Latin America and Caribbean, and the lowest in North Africa/West Asia/Eastern Europe with 4.08%. At the country level, the lowest rates of IPV among pregnant women were found in Tajikistan, Armenia, and Burkina Faso (1.01%, 1.14%, and 1.18%, respectively), and the highest rates in Papa New Guinea, South Africa, and Afghanistan (16.24%, 14.97%, and 14.75%, respectively). These findings are also illustrated geographically in Fig. 1 (Global map of intimate partner violence during pregnancy).

**Prevalence of help-seeking**

The percentages of help-seeking, conditional on having experienced IPV during pregnancy, are presented in Table 1 (Prevalence of IPV during pregnancy and help-seeking behavior by country, weighted). On a global scale, only half of women (51.87%) sought help for IPV during pregnancy from any source, ranging from 39.02% in Asia and Melanesia to 63.18% in sub-Saharan Africa. On a national scale, the lowest help-seeking rates were found in Senegal, Afghanistan, and Nepal (<0.01%, 34.24% and 34.50%, respectively), and the highest in Liberia, Tanzania, and Kyrgyz Republic (86.27%, 75.03% and 73.07%, respectively).

Globally, most survivors of IPV sought help from **informal/non-institutional** sources (44.02%), with a regional range from 35.12% in Latin America and Caribbean to 54.81% in sub-Saharan Africa. The lowest informal help-seeking rates were observed in Senegal, Guatemala, and Timor-Leste (<0.01%, 27.40%, and 28.68%, respectively), whereas the highest in Liberia (80.24%), Armenia (72.22%), and Ghana (71.35%). Among informal sources, most women sought help for IPV from their family, with a global prevalence of 30.78%, ranging from <0.01% in Senegal and 7.89% in Ethiopia to 67.37% in Kyrgyz Republic and 67.12% in Armenia. Respondents’ second most common informal source of help were in-laws, with 17.38% globally, and national percentages of <0.01% and 0.58% up to 42.93% in Senegal, Nepal, and Sierra Leone, respectively. Globally, few women sought help from neighbors (10.37%), which was found to be less common in Senegal (<0.01%) and Pakistan (0.62%), and most common in Rwanda (47.13%). On a global scale, rates of help-seeking from friends were very low with 5.90%, with the lowest national estimates of <0.01% in Armenia, Namibia, and Senegal, and the highest in Sierra Leone (23.83%).

**Fig. 2:**
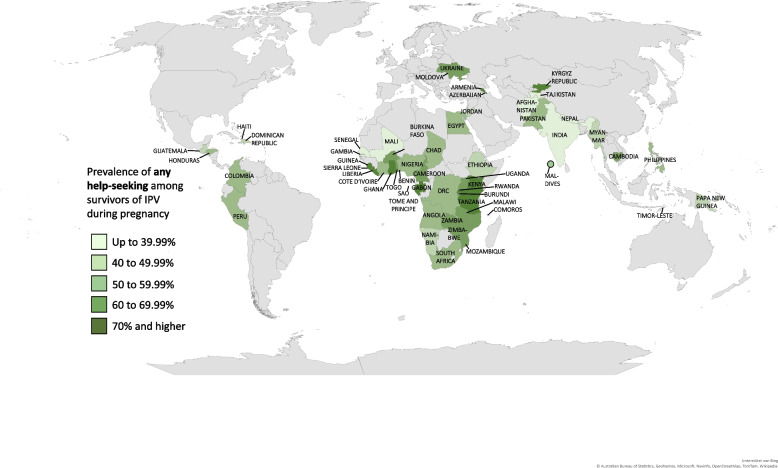
Global map of any help-seeking among survivors of IPV during pregnancy Notes: Global map of the prevalence distribution of any help-seeking among survivors of IPV during pregnancy, DSH 2005 – 2020. Results are divided into the adjacent five different colour ranges. Results are derived from Table 1

**Fig. 3:**
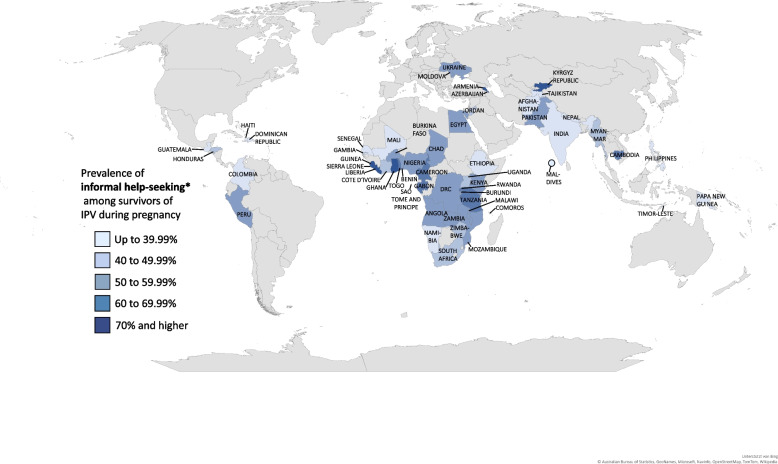
Global map of informal help-seeking among survivors of IPV during pregnancy Notes: Global map of the prevalence distribution of informal help-seeking among survivors of IPV during pregnancy, DSH 2005 – 2020. Results are divided into the adjacent five different colour ranges. Results are derived from Table 1. *Informal help-seeking sources include family, in-laws, neighbors and friends

**Fig. 4:**
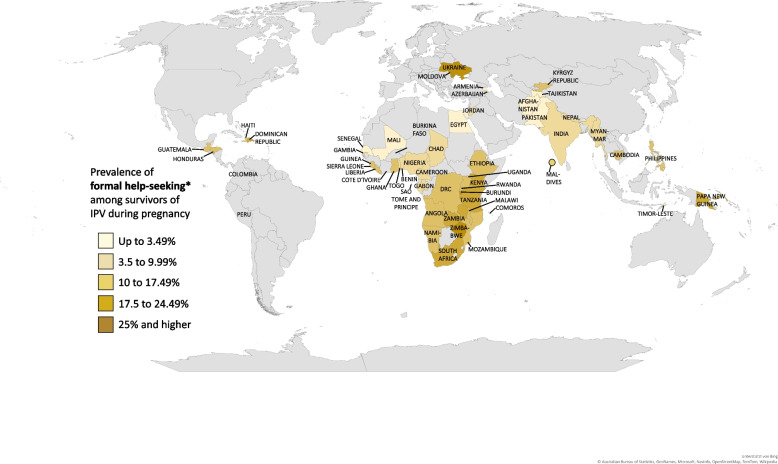
Global map of formal help-seeking among survivors of IPV during pregnancy Notes: Global map of the prevalence distribution of formal help-seeking among survivors of IPV during pregnancy, DSH 2005 – 2020. Results are divided into the adjacent five different colour ranges. Results are derived from Table 1. *Formal help-seeking sources include police, medical professionals, lawyers, social service organizations, and religious leaders

On a global scale, only one in ten women (10.45%) sought help in response to IPV during pregnancy from **formal/institutional** sources, ranging regionally from 4.66% in Asia and Melanesia to 16.21% in Latin America and Caribbean. The lowest national estimates for formal help-seeking were reported in Armenia, Senegal (both <0.01%), Pakistan (0.93%), and Mali (0.99%), whereas the highest in Moldova, Ukraine, and Burundi (30.11%, 27.57%, 26.74%, respectively). Among formal sources, the majority of women sought help from the police (5.95%), with the lowest prevalence in Armenia, Myanmar, Mali, Comoros, and Senegal (all <0.01%), and the highest in Moldova and Ukraine (27.16% and 26.12%, respectively). The second most common institutional source from which IPV survivors sought help, were social services, including lawyers, social service organizations and religious leaders, with a global mean of 4.55%, and national differences between <0.01% in Maldives, Tajikistan, Armenia, and Senegal, and 23.35% in Burundi. Medical professionals were the least common formal source from which respondents sought help for IPV during pregnancy, with a global mean of 1.12%, the lowest rate of <0.01% in Myanmar, Timor-Leste, Armenia, Egypt, Jordan, Benin, Comoros, Namibia, Sao Tome and Principe, and Senegal, and the highest rates in Moldova (5.78%) and Liberia (5.11%). These findings are also illustrated geographically in Fig. 2 (Global map of any help-seeking among survivors of IPV during pregnancy), Fig. 3 (Global map of informal help-seeking among survivors of IPV during pregnancy), and Fig. 4 (Global map of formal help-seeking among survivors of IPV during pregnancy).

**Table 1:** Prevalence of IPV during pregnancy and help-seeking behavior by country, weighted

Country by region	DHS Survey year	Sample size	Experience of IPV during pregnancy (%)	Among survivors of IPV during pregnancy									
				Sought any help (%)	Sought informal help from					Sought formal help from			
					any informal help (%)	family (%)	in-laws (%)	neighbor (%)	friends (%)	any formal help (%)	police (%)	doctor (%)	social services (%)
**Asia and Melanesia**													
Afghanistan	2015	21,302	14.75	34.24	33.95	26.40	15.79	11.02	1.63	1.43	0.14	0.08	1.32
Cambodia	2014-15	3,498	3.50	69.54	64.8	40.41	10.77	25.73	3.26	14.13	12.84	0.93	1.96
India	2015-16	66,013	3.42	34.73	32.66	24.05	10.56	5.78	4.20	3.52	1.92	0.49	1.52
Kyrgyz Republic	2012	4,831	5.92	73.07	71.10	67.37	25.55	8.46	3.28	10.71	8.58	2.14	2.25
Maldives	2016-17	3,388	2.95	50.22	39.74	25.94	2.27	1.79	17.69	10.71	9.56	1.15	<0.01
Myanmar	2015-16	3,425	1.54	47.65	41.94	21.51	5.34	17.51	2.68	3.50	<0.01	<0.01	3.50
Nepal	2016	3,826	4.77	34.50	33.04	23.71	0.58	12.77	9.49	4.11	3.77	0.26	0.53
Pakistan	2017-18	4,084	7.07	52.40	52.28	42.59	23.64	0.62	0.44	0.93	0.22	0.23	0.57
Papua New Guinea	2016-18	3,944	16.24	48.48	39.26	30.48	8.94	8.27	5.71	19.89	9.70	1.13	11.06
Philippines	2017	13,215	1.92	52.28	37.90	24.93	3.81	10.66	4.60	11.60	5.72	0.79	7.31
Timor-Leste	2016	3,694	1.52	35.39	28.68	27.25	3.12	1.43	0.75	11.61	11.61	<0.01	2.26
Tajikistan	2017	5,313	1.01	36.70	32.81	19.85	9.18	3.62	1.88	2.70	2.70	1.47	<0.01
Total Asia and Melanesia		136,533	5.56	39.02	36.65	28.10	13.08	8.87	3.26	4.66	2.44	0.43	2.34
**Latin America and Caribbean**													
Colombia	2015-16	24,890	8.41	54.88	34.62	19.60	6.46	-	15.92^b^	-	-	-	-
Dominican Republic	2013	5,801	6.75	48.09	30.65	24.35	5.13	-	5.33	21.09	15.77	1.90	3.94
Guatemala	2014-15	6,510	5.73	46.32	27.40	22.21	4.68	-	2.90	11.24	9.00	-	2.23
Haiti	2016-17	4,322	4.30	45.76	39.46	26.58	18.63	11.00	13.14	11.22	4.76	0.57	5.90
Honduras	2011-12	12,494	7.18	54.43	40.67	28.81	4.95	9.02	6.55	17.15	12.48	2.08	4.32
Peru^a^	2012	13,483	9.49	53.95	50.00	26.13	12.98	16.94	2.82^b^	-	-	-	-
Total Latin America and Caribbean		54,017	7.34	52.95	35.12	22.60	6.39	9.36	11.58	16.21	11.71	1.84	3.98
**North Africa/West Asia/Eastern Europe**													
Armenia	2016	3,539	1.14	72.22	72.22	67.12	17.14	4.95	<0.01	<0.01	<0.01	<0.01	<0.01
Azerbaijan	2006	4,301	4.05	47.75	42.88	33.64	22.67	4.47	2.61	10.79	8.88	2.61	1.30
Egypt	2014	6,692	6.21	59.73	59.26	44.43	20.36	6.38	1.11	1.30	1.30	<0.01	0.43
Jordan	2017-18	6,852	1.84	44.31	43.19	32.29	14.90	1.83	1.78	5.24	3.96	<0.01	3.07
Moldova	2005	4,593	6.59	68.38	53.94	42.07	22.66	10.53	2.74	30.11	27.16	5.78	4.07
Ukraine	2007	2,453	3.83	69.12	56.56	49.55	33.98	5.67	13.93	27.57	26.12	2.84	2.98
Total North Africa/West Asia/		28,430	4.08	59.64	54.07	42.13	21.56	6.54	2.74	12.24	10.98	2.03	1.94
Eastern Europe													
**Sub-Saharan Africa**													
Angola	2015-16	7,669	4.71	55.73	51.60	31.29	18.10	14.81	7.04	10.72	6.82	0.90	5.09
Benin	2017-18	4,488	2.61	54.06	50.15	34.99	18.34	5.35	2.87	4.63	1.27	<0.01	4.63
Burkina Faso	2010	10,009	1.18	65.15	51.48	30.52	27.67	-	0.19	-	-	-	-
Burundi	2016-17	7,366	8.83	64.53	54.11	25.83	29.03	26.99	9.56	26.74	4.63	1.31	23.35
Cameroon	2018-19	4,690	5.20	67.31	64.15	51.07	26.17	13.03	11.11	7.67	3.07	2.65	3.27
Chad	2014-15	3,810	5.70	55.07	52.60	43.42	13.95	6.19	1.39	8.43	2.10	1.06	5.26
Comoros	2012	2,529	1.67	35.12	35.12	21.05	15.62	17.81	9.08	2.92	<0.01	<0.01	2.92
Congo Democratic Republic	2013-14	5,686	10.65	57.92	54.62	31.07	31.09	19.02	9.91	11.05	1.34	1.33	8.82
Ethiopia	2016	4,720	3.27	46.30	39.00	7.98	12.00	19.02	3.69	14.62	2.49	1.88	10.25
Gabon	2012	4,147	8.73	70.17	64.51	37.95	24.61	21.99	3.47	9.22	4.86	1.61	3.10
Gambia	2019-20	1,953	5.63	43.52	41.03	33.44	12.16	9.31	2.67	4.88	2.13	2.86	3.51
Ghana	2008	1,836	2.76	83.45	71.35	48.47	26.77	1.75	5.19	11.39	7.31	1.18	5.76
Ivory Coast	2011-12	5,005	2.94	55.60	40.27	22.87	19.36	-	2.76	-	-	-	-
Kenya	2014	4,515	8.03	71.75	59.65	42.03	27.24	8.85	3.90	22.78	12.44	3.27	8.62
Liberia	2019-20	2,331	4.12	86.27	80.24	52.75	39.28	12.60	9.27	14.87	4.95	5.11	6.10
Malawi	2015-16	5,406	3.73	69.90	55.11	42.98	31.90	5.94	6.34	14.98	11.23	0.92	3.55
Mali	2018	3,356	5.45	31.49	30.94	19.86	19.22	0.94	0.30	0.99	<0.01	0.13	0.86
Mozambique	2015 AIS	3,355	3.70	63.13	58.92	39.94	31.42	3.06	8.28	15.55	10.37	0.68	5.19
Namibia	2013	1,448	4.13	44.56	22.96	15.19	7.78	2.85	<0.01	11.46	8.61	<0.01	2.85
Nigeria	2018	8,910	3.59	57.06	52.81	40.72	22.37	2.18	3.31	4.42	0.36	0.69	3.59
Rwanda	2014-15	1,907	8.08	73.31	69.04	16.94	21.43	47.13	6.26	15.55	9.51	0.60	7.18
Sao Tome and Principe	2008-09	1,727	6.17	62.67	54.41	37.55	14.63	20.92	5.30	13.84	5.56	<0.01	8.29
Senegal	2019	1,468	2.54	<0.01	<0.01	<0.01	<0.01	<0.01	<0.01	<0.01	<0.01	<0.01	<0.01
Sierra Leone	2019	4,055	4.08	72.95	70.58	63.20	42.93	19.20	23.83	17.25	8.19	0.83	8.80
South Africa	2016	4,003	14.97	59.05	47.23	36.24	19.89	11.28	12.2	22.94	19.99	2.95	3.71
Tanzania	2015-16	7,596	7.73	75.03	60.35	45.46	39.16	12.31	4.30	18.48	9.91	0.78	10.10
Togo	2013-14	5,368	6.64	69.39	64.56	44.59	31.33	6.31	3.25	6.38	2.04	0.48	4.36
Uganda	2016	7,536	9.49	62.28	49.35	33.80	26.14	6.54	5.40	16.75	12.62	3.41	3.56
Zambia	2018-19	7,358	4.68	64.76	53.56	40.80	37.29	5.43	4.40	23.35	17.21	3.14	6.28
Zimbabwe	2015	5,800	4.64	64.86	48.65	35.70	27.59	2.85	5.01	26.27	22.46	0.47	6.14
Total Sub-Saharan Africa		140,047	5.34	63.18	54.81	36.17	27.12	12.74	6.00	14.83	7.36	1.55	7.48
**Total**		359,027	5.63	51.87	44.02	30.78	17.38	10.37	5.90	10.45	5.95	1.12	4.55

All variables are expressed as proportions (in %), apart from sample size. All results are weighted. “Doctor” include all medical professionals. “Social services” include lawyer, social service organization and religious leader. **–** Data not available. ^**a**^For Peru, only unweighted data was available, hence Peru’s data is excluded from regional and total weighted estimates. ^**b**^For Colombia, data was only available for “Male friend”, and for Peru, data was only available for “Female friend”

**Associations of help-seeking**

Table 2 (Logistic Regression Odds Ratios (OR) of any help-seeking for women experiencing IPV during pregnancy, by region, adjusted and weighted), Table 3 (Logistic Regression Odds Ratios (OR) of informal/non-institutional help-seeking for women experiencing IPV during pregnancy, by region, adjusted and weighted), and Table 4 (Logistic Regression Odds Ratios (OR) of formal/institutional help-seeking for women experiencing IPV during pregnancy, by region, adjusted and weighted) present the logistic regression results assessing characteristics associated with IPV survivors’ help-seeking from any source, listed by informal and formal sources, respectively.

**Women’s individual characteristics**

Women’s age between 35 and 49 years was significantly associated with lower odds of seeking any or informal help in Asia and Melanesia, and sub-Saharan Africa, whereas women of the same age group were more likely to seek formal help in Latin America and Caribbean and sub-Saharan Africa, compared to women aged 15 to 19 years. Respondent’s age between 15 and 19 years at first cohabitation significantly fostered IPV survivors help-seeking for formal help in Latin America and Caribbean (up to aOR = 1.61 (CI = 1.17; 2.22)); being 20 years and older compared to an age under 15 years at first cohabitation was associated with decreased help-seeking (lowest aOR for any help in Latin America and Caribbean was of 0.36 (CI = 0.22; 0.57)). However, the total estimates revealed that women who cohabitate at older ages (25 years and older) are more likely to seek all forms of help (highest aOR of 1.72 (CI = 1.18; 2.50) for any help in Total). Consistently in all regions, and for all sources of help, married women were less likely to seek help compared to those previously married (aORs ranging from 0.01 (CI = 0.01; 0.05) for formal help in North Africa/West Asia/Eastern Europe to 0.48 (CI = 0.38; 0.62) for informal help in Latin America and Caribbean). Compared to no education, having primary education (up to aOR = 2.83 (CI = 1.80; 4.45) in Asia and Melanesia), and secondary or higher education significantly facilitated women’s help-seeking (with the highest aOR of 3.98 (CI = 2.52; 6.29) for formal help in Asia and Melanesia). Being employed was positively associated with help-seeking in two regions compared to the non-working respondents (aORs ranging from 1.17 (CI = 1.00; 1.37) for informal help in Asia and Melanesia to 1.56 (CI = 1.23; 1.98) for formal help in sub-Saharan Africa). If a woman earned more than her husband, she was more likely to seek help in response to IPV during pregnancy (up to aOR = 5.53 (CI = 1.15; 26.66) for any and informal help in North Africa/West Asia/Eastern Europe), whereas if her husband/partner earned more than her, she was less likely to seek help from family, neighbors, and friends in two regions (with a range of aORs from 0.58 (CI = 0.33; 0.99) in Asia and Melanesia to 0.65 (CI = 0.44; 0.95) in Latin America and Caribbean), compared to women earning about the same as their husbands. Exposure to mass media - at least once a week - was positively associated with help-seeking in three regions: reading newspapers or magazines, listening to radio (up to aOR = 2.23 (CI = 1.19; 4.16) for formal help and up to aOR = 1.58 (CI = 1.35; 1.85) for any help, both in Asia and Melanesia, respectively), and watching television was associated with higher odds of help-seeking (with highest aOR of 1.68 (CI = 1.15; 2.44) for formal help in Latin America and Caribbean). However, IPV survivors who watched television regularly were also less likely to seek formal help in Asia and Melanesia (aOR = 0.44; CI = 0.26; 0.73), and any help in sub-Saharan Africa (aOR = 0.86; CI = 0.74; 1.00). When an IPV survivor indicated that she would have preferred to be pregnant at an earlier point in life compared to later, she was less likely to seek help (with lowest aOR of 0.17 (CI = 0.04; 0.75) for any and informal help in North Africa/West Asia/Eastern Europe).

**Partner/family characteristics**

Generally, having an older partner (aged 41 years and above) compared to a partner aged under 21 years was negatively associated with help-seeking in all regions (with lowest aOR of 0.37 (CI = 0.21; 0.68) for age 41-50 for any help in North Africa/West Asia/Eastern Europe), whereas partner’s younger age of 21-40 years facilitated women’s help-seeking in two regions (with highest aOR of 1.51 (CI = 1.01; 2.24) for any help in Latin America and Caribbean). Partner’s primary, and secondary or higher education compared to no education was associated with higher odds of help-seeking in two regions (with highest aORs of 1.56 (CI = 1.02; 2.36) for formal help and 1.28 (CI = 1.03; 1.58) for any help, both in Asia and Melanesia, respectively), with the exception of North Africa/West Asia/Eastern Europe where partner’s secondary or higher education was associated with lower odds for women seeking help (aOR = 0.52 (CI = 0.31; 0.88) for any help). When the partner was working, IPV survivors were less likely to seek help in three regions compared to having a non-working partner (with lowest aOR = 0.29 (CI = 0.18; 0.46) for formal help in Asia and Melanesia). Partner’s alcohol consumption fostered women to seek help for IPV during pregnancy in three regions compared to having partners never drinking alcohol (with highest aORs of 7.01 (CI = 3.95; 12.44) for often, and 2.49 (CI = 1.39; 4.47) for sometimes drinking alcohol, both for formal help in sub-Saharan Africa). Further family factors facilitating women’s help-seeking for IPV in all regions included an increasing number of living children (up to aOR = 1.96 (CI = 1.42; 2.71) for formal help in North Africa/West Asia/Eastern Europe), women’s father abused her mother (with highest aOR of 3.85 (CI = 3.36; 4.42) for informal help in Asia and Melanesia), and being afraid of partner most of the time or sometimes (with highest aORs of 14.38 (CI = 9.28; 22.29) in North Africa/West Asia/Eastern Europe, and 2.57 (CI = 2.26; 2.94) in sub-Saharan Africa, both for informal help, respectively).

**Community/societal characteristics**

Respondents with rural residences compared to urban residences were less likely to seek help in response to IPV from formal institutions (lowest aOR = 0.50 (CI = 0.33; 0.76) for formal help in Latin America and Caribbean), but simultaneously were more likely to reach out to informal support networks (up to aOR = 1.53 (CI = 1.07; 2.19) for informal help in North Africa/West Asia/Eastern Europe), except for Latin America and Caribbean where rural residence was generally associated with lower odds of help-seeking.

Globally, the odds of help-seeking in response to IPV decreased in countries with middle wealth index compared to poorer or poorest wealth index and increased in those within the highest wealth quintile, but results varied to a large extent by region. Women with a higher economic status were more likely to seek all forms of help in Asia and Melanesia (highest aOR = 1.73 (CI = 1.16; 2.57) for any help), and formal help in sub-Saharan Africa (aOR = 1.73 (CI = 1.36; 2.22)). By contrast, an increasing wealth index was negatively associated with help-seeking in three regions (lowest aORs of 0.09 (CI = 0.01; 0.84) in North Africa/West Asia/Eastern Europe, 0.48 (CI = 0.30; 0.77) in Latin America and Caribbean, both for formal help, and 0.75 (CI = 0.66; 0.86) in sub-Saharan Africa for informal help).

If respondents shared decision-making with their partner, or partner alone had the final say in decision-making, women were less likely to seek help in three regions (lowest aORs of 0.70 (CI = 0.52; 0.94) and 0.48 (CI = 0.23; 1.00), both for informal help in Latin America and Caribbean, respectively), apart from Asia and Melanesia where decision-making of partner alone encouraged woman to seek any or informal help (highest aOR = 1.54 (CI = 1.23; 1.93) for informal help), compared to decision-making of woman alone.

Consistently in all regions and for all sources of help, all controlling behaviors by a partner facilitated IPV survivor’s help-seeking (except for formal help in North Africa/West Asia/Eastern Europe).

In general, justification of physical violence against wives was associated with lower odds of seeking help in response to IPV during pregnancy.

Facing “big problems” when accessing health care generally fostered women’s help-seeking in all regions, but simultaneously hindered IPV survivors in North Africa/West Asia/Eastern Europe and sub-Saharan Africa from reaching out to formal institutions.

**Reasons for not seeking help**

For Dominican Republic, Peru, Burkina Faso, and Ivory Coast, data was also available concerning the main reasons women never sought help, as presented in Table 5 (Descriptive statistics of reasons for not seeking help among women experiencing IPV during pregnancy, by country, weighted). The main reasons indicated by IPV survivors were that help was of no use (37.12% in Ivory Coast and 32.72% in Burkina Faso), that the experience of violence was part of life (31.64% in Burkina Faso and 12.71% in Ivory Coast), that they did not know where to go to (18.95% in Peru and 14.65% in Ivory Coast), that they were afraid of further beatings (22.55% in Dominican Republic and 21.32% in Peru), afraid of getting the partner in trouble (13.67% in Dominican Republic and 10.79% in Peru), or embarrassed (22.63% in Peru and 12.14% in Dominican Republic).

**Table 2:** Logistic Regression Odds Ratios (OR) of any help-seeking for women experiencing IPV during pregnancy, by region, adjusted and weighted

	Asia and Melanesia			Latin America and Caribbean^a^			North Africa/ West Asia/Eastern Europe			Sub-Saharan Africa			Total^a^		
	aOR	95% CI	*p*-value	aOR	95% CI	*p*-value	aOR	95% CI	*p*-value	aOR	95% CI	*p*-value	aOR	95% CI	*p*-value
***Women’s characteristics***															
**Woman’s age (years)**															
15-19															
20-24	1.04	0.67; 1.61	0.877	1.18	0.68; 2.03	0.556	1.42	0.52; 3.87	0.488	1.25	0.99; 1.58	0.065	1.12	0.92; 1.36	0.253
25-29	0.92	0.59; 1.43	0.709	1.32	0.77; 2.25	0.313	0.55	0.19; 1.57	0.264	1.11	0.87; 1.41	0.387	0.96	0.79; 1.17	0.701
30-34	0.96	0.61; 1.50	0.851	1.39	0.81; 2.38	0.233	0.71	0.24; 2.07	0.530	0.95	0.74; 1.23	0.709	0.88	0.72; 1.07	0.210
35-39	0.80	0.50; 1.28	0.358	**1.83**	**1.05; 3.19**	0.034*	0.70	0.23; 2.11	0.524	**0.75**	**0.57; 0.99**	0.044*	**0.75**	**0.60; 0.93**	0.008*
40-44	**0.62**	**0.39; 1.00**	0.049*	1.42	0.77; 2.59	0.259	0.50	0.16; 1.58	0.237	**0.66**	**0.50; 0.88**	0.004*	**0.60**	**0.48; 0.75**	<0.001**
45-49	0.67	0.41; 1.08	0.100	1.34	0.74; 2.44	0.336	0.38	0.12; 1.21	0.102	**0.68**	**0.50; 0.92**	0.012*	**0.62**	**0.49; 0.77**	<0.001**
**Age at first cohabitation (years)**															
<15															
15-19	**1.27**	**1.06; 1.52**	0.010*	1.13	0.94; 1.37	0.196	0.99	0.72; 1.37	0.964	**1.12**	**1.03; 1.21**	0.008*	**1.41**	**1.09; 1.83**	0.009*
20-24	0.86	0.70; 1.05	0.130	**0.57**	**0.42; 0.79**	<0.001**	0.82	0.58; 1.15	0.257	0.99	0.88; 1.11	0.838	**1.37**	**1.03; 1.84**	0.032*
25+	1.08	0.84; 1.39	0.545	**0.36**	**0.22; 0.57**	<0.001**	1.26	0.79; 2.01	0.329	0.89	0.72; 1.09	0.255	**1.72**	**1.18; 2.50**	0.005*
**Marital status**															
Previously married															
Currently married	**0.16**	**0.11; 0.23**	<0.001**	**0.43**	**0.35; 0.52**	<0.001**	**0.14**	**0.09; 0.22**	<0.001**	**0.39**	**0.35; 0.44**	<0.001**	**0.33**	**0.30; 0.36**	<0.001**
**Woman’s education**															
No education															
Primary	**1.33**	**1.00; 1.77**	0.050*	**1.45**	**1.09; 1.92**	0.011*	1.10	0.51; 2.36	0.812	**1.54**	**1.38; 1.72**	<0.001**	**1.64**	**1.45; 1.87**	<0.001**
Secondary or higher	**1.39**	**1.09; 1.78**	0.008*	**1.65**	**1.20; 2.27**	0.002*	1.73	0.88; 3.44	0.114	**1.23**	**1.06; 1.42**	0.005*	**1.58**	**1.37; 1.84**	<0.001**
**Woman is working**															
No															
Yes	**1.23**	**1.06; 1.42**	0.007*	1.13	0.94; 1.36	0.204	1.23	0.83; 1.82	0.311	**1.49**	**1.33; 1.66**	<0.001**	**1.39**	**1.30; 1.49**	<0.001**
**Who earns more**															
About the same															
Husband/partner earns more	0.87	0.56; 1.36	0.540	0.81	0.57; 1.15	0.242	3.42	0.77; 15.27	0.107	1.14	0.91; 1.42	0.265	**1.30**	**1.10; 1.54**	0.002*
Woman earns more than him	**1.83**	**1.20; 2.80**	0.005*	1.11	0.71; 1.74	0.632	**5.53**	**1.15; 26.66**	0.033*	**1.47**	**1.10; 1.98**	0.010*	**1.55**	**1.24; 1.93**	<0.001**
**Exposure to mass media (at least once a week)**															
Reading newspaper or magazine	**1.44**	**1.11; 1.87**	0.006*	1.00	0.77; 1.29	0.990	0.69	0.39; 1.23	0.212	**1.20**	**1.02; 1.40**	0.028*	**1.12**	**1.01; 1.23**	0.030*
Listening to radio	**1.58**	**1.35; 1.85**	<0.001**	1.06	0.85; 1.34	0.600	0.99	0.63; 1.57	0.979	**1.12**	**1.01; 1.24**	0.038*	**1.17**	**1.09; 1.26**	<0.001**
Watching television	**1.27**	**1.02; 1.60**	0.035*	1.29	1.00; 1.67	0.054	0.98	0.45; 2.11	0.950	**0.86**	**0.74; 1.00**	0.050*	**0.87**	**0.79; 0.95**	0.003*
**Current pregnancy wanted**															
Later															
Then	1.25	0.65; 2.41	0.501	0.82	0.30; 2.23	0.700	**0.17**	**0.04; 0.75**	0.019*	**0.74**	**0.56; 0.99**	0.041*	0.80	0.63; 1.02	0.068
Not at all	0.93	0.38; 2.25	0.864	2.37	0.85; 6.63	0.100	0.73	0.06; 8.63	0.804	1.40	0.90; 2.18	0.133	1.09	0.76; 1.56	0.640
***Partner/family characteristics***															
**Partner’s age (years)**															
<21															
21-30	1.01	0.84; 1.22	0.890	**1.51**	**1.01; 2.24**	0.043*	0.68	0.32; 1.44	0.309	1.04	0.91; 1.20	0.536	1.07	0.97; 1.19	0.180
31-40	0.97	0.85; 1.10	0.620	1.07	0.86; 1.34	0.543	0.91	0.62; 1.33	0.626	**1.13**	**1.02; 1.26**	0.020*	1.04	0.96; 1.12	0.309
41-50	0.98	0.82; 1.16	0.798	0.85	0.65; 1.11	0.237	**0.37**	**0.21; 0.68**	0.001*	0.98	0.86; 1.11	0.737	0.96	0.87; 1.05	0.360
51-60	1.15	0.90; 1.49	0.267	**0.41**	**0.17; 0.99**	0.048*	1.33	0.77; 2.30	0.306	**0.77**	**0.64; 0.93**	0.007*	0.94	0.82; 1.08	0.372
>60	**0.48**	**0.24; 0.97**	0.040*	0.91	0.47; 1.75	0.778	1.05	0.48; 2.34	0.896	0.79	0.59; 1.06	0.113	0.82	0.66; 1.04	0.101
**Partner’s education**															
No education															
Primary	**1.47**	**1.12; 1.93**	0.006*	1.13	0.85; 1.50	0.411	0.99	0.58; 1.69	0.969	**1.54**	**1.34; 1.77**	<0.001**	**1.59**	**1.37; 1.85**	<0.001**
Secondary or higher	**1.28**	**1.03; 1.58**	0.024*	1.01	0.68; 1.48	0.973	**0.52**	**0.31; 0.88**	0.015*	**1.14**	**1.02; 1.28**	0.025*	**1.22**	**1.04; 1.42**	0.012*
**Partner is working**															
No															
Yes	**0.55**	**0.38; 0.79**	0.001*	0.52	0.24; 1.11	0.089	1.90	0.95; 3.78	0.069	**0.66**	**0.45; 0.96**	0.029*	**0.75**	**0.59; 0.95**	0.019*
**Partner drinks alcohol**															
Never															
Often	**2.52**	**1.78; 3.58**	<0.001**	**1.65**	**1.25; 2.19**	<0.001**	0.38	0.10; 1.53	0.173	**3.71**	**2.97; 4.64**	<0.001**	**3.86**	**3.20; 4.66**	<0.001**
Sometimes	**1.46**	**1.05; 2.04**	0.025*	1.17	0.92; 1.50	0.202	0.39	0.10; 1.51	0.173	**1.51**	**1.21; 1.89**	<0.001**	**1.28**	**1.07; 1.55**	0.008*
**Number of living children**	**1.18**	**1.14; 1.22**	<0.001**	**1.09**	**1.03; 1.15**	0.005*	1.07	0.96; 1.20	0.213	**1.13**	**1.10; 1.16**	<0.001**	**1.15**	**1.13; 1.18**	<0.001**
**Women’s father abused her mother**															
No															
Yes	**3.69**	**3.23; 4.21**	<0.001**	**2.17**	**1.79; 2.61**	<0.001**	**3.07**	**2.19; 4.31**	<0.001**	**2.17**	**1.98; 2.38**	<0.001**	**2.71**	**2.53; 2.90**	<0.001**
**Afraid of partner**															
Never															
Most of the time	**7.63**	**6.08; 9.59**	<0.001**	**3.70**	**2.87; 4.78**	<0.001**	**14.09**	**9.12; 21.78**	<0.001**	**7.39**	**6.54; 8.36**	<0.001**	**6.50**	**5.93; 7.14**	<0.001**
Sometimes	**1.86**	**1.47; 2.34**	<0.001**	1.05	0.85; 1.29	0.644	**2.29**	**1.50; 3.48**	<0.001**	**2.52**	**2.23; 2.86**	<0.001**	**1.85**	**1.69; 2.04**	<0.001**
***Community/societal characteristics***															
**Place of residence**															
Urban															
Rural	**1.20**	**1.00; 1.43**	0.044*	**0.64**	**0.52; 0.80**	<0.001**	**1.52**	**1.06; 2.18**	0.021*	1.05	0.94; 1.18	0.393	**1.27**	**1.01; 1.61**	0.043*
**Wealth index**															
Poorer or Poorest															
Middle	1.11	0.78; 1.60	0.553	1.00	0.79; 1.25	0.970	1.01	0.66; 1.53	0.979	**0.86**	**0.76; 0.96**	0.008*	**0.89**	**0.82; 0.97**	0.009*
Richer or Richest	**1.73**	**1.16; 2.57**	0.007*	**0.64**	**0.49; 0.82**	<0.001**	1.24	0.79; 1.94	0.353	**0.80**	**0.70; 0.91**	<0.001**	**1.22**	**1.05; 1.43**	0.011*
**Decision making**															
Woman alone															
Woman and partner	**0.80**	**0.66; 0.97**	0.024*	**0.72**	**0.57; 0.93**	0.011*	0.80	0.49; 1.31	0.375	**0.73**	**0.63; 0.84**	<0.001**	**0.82**	**0.72; 0.93**	0.003*
Partner alone	**1.48**	**1.20; 1.83**	<0.001**	0.71	0.50; 1.01	0.059	0.89	0.51; 1.58	0.699	**0.86**	**0.74; 1.00**	0.043*	**1.22**	**1.05; 1.41**	0.009*
**Controlling behavior: Partner...**															
jealous if respondent talks with other men/women	**4.23**	**3.64; 4.90**	<0.001**	**3.04**	**2.48; 3.73**	<0.001**	**2.38**	**1.63; 3.50**	<0.001**	**2.99**	**2.69; 3.32**	<0.001**	**3.52**	**3.25; 3.80**	<0.001**
accuses respondent of unfaithfulness	**4.20**	**3.66; 4.83**	<0.001**	**3.78**	**3.13; 4.56**	<0.001**	**3.93**	**2.59; 5.96**	<0.001**	**3.13**	**2.85; 3.43**	<0.001**	**3.67**	**3.42; 3.94**	<0.001**
does not permit respondent to meet female/male friends	**2.68**	**2.34; 3.07**	<0.001**	**3.29**	**2.73; 3.96**	<0.001**	**3.20**	**2.19; 4.69**	<0.001**	**2.86**	**2.60; 3.14**	<0.001**	**2.90**	**2.71; 3.11**	<0.001**
tries to limit respondent’s contact with family	**3.38**	**2.94; 3.89**	<0.001**	**3.19**	**2.61; 3.90**	<0.001**	**4.14**	**2.89; 5.93**	<0.001**	**3.15**	**2.84; 3.48**	<0.001**	**3.30**	**3.06; 3.56**	<0.001**
insists on knowing where respondent is	**3.44**	**3.02; 3.93**	<0.001**	**2.59**	**2.14; 3.15**	<0.001**	**2.53**	**1.85; 3.46**	<0.001**	**2.54**	**2.31; 2.79**	<0.001**	**2.94**	**2.75; 3.15**	<0.001**
**Wife beating justified if wife**															
goes out without telling husband	0.91	0.75; 1.10	0.349	0.66	0.37; 1.19	0.170	0.99	0.57; 1.72	0.971	**0.86**	**0.74; 1.00**	0.044*	**0.93**	**0.87; 0.99**	0.015*
neglects the children	0.86	0.65; 1.12	0.254	0.85	0.50; 1.44	0.533	0.64	0.38; 1.10	0.106	**0.86**	**0.74; 0.99**	0.039*	**0.93**	**0.87; 0.98**	0.011*
argues with husband	**0.74**	**0.56; 0.98**	0.033*	0.57	0.28; 1.16	0.121	**0.51**	**0.28; 0.92**	0.025*	**0.84**	**0.72; 0.98**	0.022*	**0.90**	**0.84; 0.96**	0.002*
refuses to have sex with husband	0.87	0.64; 1.18	0.365	**0.63**	**0.41; 0.97**	0.036*	0.54	0.29; 1.01	0.054	**0.79**	**0.68; 0.92**	0.002*	0.99	0.83; 1.17	0.869
burns the food	0.77	0.58; 1.03	0.083	0.54	0.25; 1.18	0.122	**0.32**	**0.15; 0.70**	0.004*	**0.89**	**0.82; 0.98**	0.014*	**0.89**	**0.82; 0.96**	0.002*
**Health-seeking barriers (Following is a “big problem”)**															
getting permission to go	**2.47**	**2.03; 3.00**	<0.001**	**1.70**	**1.23; 2.35**	0.001*	**1.75**	**1.12; 2.73**	0.015*	**0.82**	**0.69; 0.99**	0.038*	**2.29**	**1.97; 2.65**	<0.001**
getting money needed for treatment	**2.38**	**1.89; 3.00**	<0.001**	**3.23**	**2.06; 5.06**	<0.001**	1.26	0.87; 1.84	0.223	**1.25**	**1.13; 1.38**	<0.001**	**2.83**	**2.32; 3.46**	<0.001**
distance to health care facility	**2.47**	**1.91; 3.20**	<0.001**	**1.44**	**1.04; 2.02**	0.031*	**1.56**	**1.09; 2.22**	0.015*	0.97	0.88; 1.06	0.471	**2.25**	**1.86; 2.73**	<0.001**
not wanting to go alone	**2.40**	**1.93; 2.98**	<0.001**	**1.80**	**1.32; 2.47**	<0.001**	**1.93**	**1.39; 2.69**	<0.001**	1.00	0.89; 1.11	0.936	**2.35**	**1.99; 2.77**	<0.001**
**Sample size**		**2,556**			**1,856**			**610**			**4,050**			**9,072**	

aOR, adjusted Odds Ratio. 95% CI, 95% Confidence Interval. All results are weighted. ^**a**^ For Peru, only unweighted data was available, hence Peru’s data is excluded from regional and total weighted estimates. * p-value < 0.05. ** p-value < 0.001.

ORs were adjusted for women’s age, women’s age at first cohabitation, women’s education, women’s working status, women’s marital status, number of living children, partner’s education and working status, whether her partner drank alcohol, whether her father abused her mother, woman afraid of partner, wealth index and rural/urban residence

**Table 3:** Logistic Regression Odds Ratios (OR) of informal/non-institutional help-seeking for women experiencing IPV during pregnancy, by region, adjusted and weighted

	Asia and Melanesia			Latin America and Caribbean^a^			North Africa/West Asia/Eastern Europe			Sub-Saharan Africa			Total^a^		
	aOR	95% CI	*p*-value	aOR	95% CI	*p*-value	aOR	95% CI	*p*-value	aOR	95% CI	*p*-value	aOR	95% CI	*p*-value
***Women’s characteristics***															
**Woman’s age (years)**															
15-19															
20-24	0.99	0.63; 1.55	0.964	1.01	0.54; 1.90	0.972	1.45	0.53; 3.94	0.467	1.20	0.94; 1.54	0.135	1.07	0.88; 1.31	0.486
25-29	0.87	0.56; 1.35	0.529	1.28	0.69; 2.38	0.428	0.57	0.20; 1.62	0.292	1.08	0.85; 1.39	0.517	0.93	0.76; 1.14	0.491
30-34	0.92	0.58; 1.44	0.705	1.45	0.77; 2.70	0.247	0.74	0.25; 2.17	0.584	0.92	0.71; 1.20	0.552	0.86	0.70; 1.06	0.150
35-39	0.78	0.49; 1.26	0.313	1.59	0.83; 3.06	0.165	0.73	0.24; 2.20	0.574	**0.71**	**0.53; 0.94**	0.016*	**0.71**	**0.57; 0.88**	0.002*
40-44	**0.58**	**0.36; 0.94**	0.026*	1.28	0.62; 2.65	0.505	0.51	0.16; 1.63	0.255	**0.62**	**0.46; 0.83**	0.001*	**0.56**	**0.45; 0.70**	<0.001**
45-49	0.63	0.38; 1.02	0.061	1.18	0.59; 2.37	0.632	0.41	0.13; 1.28	0.125	**0.65**	**0.47; 0.90**	0.010*	**0.59**	**0.47; 0.75**	<0.001**
**Age at first cohabitation (years)**															
<15															
15-19	**1.23**	**1.03; 1.48**	0.023*	1.12	0.89; 1.40	0.346	1.01	0.73; 1.40	0.942	**1.11**	**1.02; 1.21**	0.013*	1.03	0.96; 1.11	0.408
20-24	0.88	0.73; 1.08	0.228	**0.54**	**0.37; 0.79**	0.001*	0.81	0.58; 1.15	0.236	0.99	0.88; 1.11	0.821	0.97	0.90; 1.06	0.557
25+	1.04	0.79; 1.36	0.790	**0.37**	**0.21; 0.66**	<0.001**	1.22	0.76; 1.96	0.399	0.87	0.69; 1.08	0.211	**1.37**	**1.01; 1.86**	0.040*
**Marital status**															
Previously married															
Currently married	**0.15**	**0.10; 0.22**	<0.001**	**0.48**	**0.38; 0.62**	<0.001**	**0.15**	**0.10; 0.22**	<0.001**	**0.42**	**0.37; 0.47**	<0.001**	**0.36**	**0.32; 0.39**	<0.001**
**Woman’s education**															
No education															
Primary	**1.23**	**1.05; 1.43**	0.010*	1.17	0.87; 1.58	0.301	1.04	0.49; 2.24	0.911	**1.51**	**1.35; 1.69**	<0.001**	**1.37**	**1.21; 1.55**	<0.001**
Secondary or higher	1.14	0.98; 1.32	0.090	1.04	0.74; 1.45	0.815	1.69	0.86; 3.34	0.129	**1.21**	**1.04; 1.41**	0.014*	**1.38**	**1.19; 1.60**	<0.001**
**Woman is working**															
No															
Yes	**1.17**	**1.00; 1.37**	0.043*	1.04	0.88; 1.24	0.625	1.24	0.84; 1.84	0.283	**1.46**	**1.30; 1.64**	<0.001**	**1.32**	**1.23; 1.42**	<0.001**
**Who earns more**															
About the same															
Husband/partner earns more	**0.58**	**0.33; 0.99**	0.048*	**0.65**	**0.44; 0.95**	0.025*	3.42	0.77; 15.27	0.107	1.07	0.84; 1.35	0.594	**1.25**	**1.05; 1.49**	0.014*
Woman earns more than him	**1.65**	**1.08; 2.52**	0.022*	1.03	0.54; 1.97	0.917	**5.53**	**1.15; 26.66**	0.033*	1.27	0.93; 1.74	0.138	**1.42**	**1.12; 1.80**	0.003*
**Exposure to mass media (at least once a week)**															
Reading newspaper or magazine	**1.40**	**1.05; 1.87**	0.021*	1.06	0.78; 1.43	0.724	0.69	0.39; 1.24	0.218	1.11	0.94; 1.31	0.215	0.97	0.83; 1.12	0.654
Listening to radio	**1.50**	**1.28; 1.75**	<0.001**	1.20	0.91; 1.58	0.208	1.00	0.63; 1.58	0.993	**1.10**	**1.01; 1.20**	0.025*	**1.12**	**1.03; 1.22**	0.006*
Watching television	**1.28**	**1.02; 1.61**	0.031*	1.17	0.86; 1.59	0.320	0.96	0.44; 2.07	0.912	**1.12**	**1.00; 1.25**	0.046*	**0.87**	**0.79; 0.96**	0.005*
**Current pregnancy wanted**															
Later															
Then	1.28	0.64; 2.53	0.485	0.45	0.14; 1.44	0.179	**0.17**	**0.04; 0.75**	0.019*	0.78	0.57; 1.05	0.100	0.82	0.63; 1.06	0.127
Not at all	0.91	0.36; 2.30	0.839	2.15	0.76; 6.06	0.150	0.73	0.06; 8.63	0.804	1.34	0.83; 2.16	0.228	1.04	0.71; 1.53	0.841
***Partner/family characteristics***															
**Partner’s age (years)**															
<21															
21-30	0.99	0.82; 1.20	0.948	1.00	0.67; 1.48	0.994	0.68	0.32; 1.43	0.304	1.04	0.90; 1.21	0.565	1.06	0.95; 1.18	0.299
31-40	0.96	0.84; 1.10	0.595	1.07	0.82; 1.40	0.617	0.92	0.63; 1.34	0.651	1.14	1.00; 1.31	0.057	1.03	0.95; 1.11	0.519
41-50	1.00	0.83; 1.19	0.986	0.80	0.57; 1.10	0.172	**0.38**	**0.21; 0.68**	0.001*	1.02	0.89; 1.17	0.802	0.98	0.89; 1.09	0.708
51-60	1.15	0.88; 1.49	0.315	0.71	0.46; 1.09	0.122	1.27	0.73; 2.22	0.392	**0.77**	**0.63; 0.94**	0.009*	0.95	0.82; 1.10	0.498
>60	0.50	0.25; 1.02	0.055	1.23	0.62; 2.44	0.552	1.06	0.48; 2.36	0.878	0.77	0.56; 1.04	0.089	0.82	0.65; 1.04	0.109
**Partner’s education**															
No education															
Primary	**1.41**	**1.08; 1.86**	0.013*	1.15	0.83; 1.60	0.394	1.00	0.58; 1.71	0.993	**1.49**	**1.29; 1.72**	<0.001**	**1.48**	**1.27; 1.72**	<0.001**
Secondary or higher	**1.24**	**1.00; 1.53**	0.048*	0.79	0.50; 1.25	0.315	**0.53**	**0.31; 0.90**	0.018*	**1.16**	**1.03; 1.30**	0.013*	**1.19**	**1.02; 1.38**	0.031*
**Partner is working**															
No															
Yes	**0.62**	**0.42; 0.93**	0.021*	**0.36**	**0.17; 0.79**	0.010*	1.91	0.95; 3.86	0.071	0.95	0.62; 1.47	0.834	0.83	0.63; 1.09	0.173
**Partner drinks alcohol**															
Never															
Often	**2.45**	**1.72; 3.50**	<0.001**	**2.26**	**1.36; 3.76**	0.002*	0.38	0.10; 1.53	0.173	**3.42**	**2.70; 4.33**	<0.001**	**3.67**	**3.01; 4.47**	<0.001**
Sometimes	**1.49**	**1.06; 2.09**	0.023*	0.71	0.45; 1.12	0.140	0.39	0.10; 1.51	0.173	**1.45**	**1.15; 1.83**	0.002*	**1.25**	**1.03; 1.52**	0.023*
**Number of living children**	**1.18**	**1.14; 1.22**	<0.001**	1.06	0.99; 1.14	0.107	1.06	0.95; 1.19	0.309	**1.12**	**1.08; 1.15**	<0.001**	**1.15**	**1.12; 1.17**	<0.001**
**Women’s father abused her mother**															
No															
Yes	**3.85**	**3.36; 4.42**	<0.001**	**2.00**	**1.60; 2.50**	<0.001**	**3.06**	**2.18; 4.29**	<0.001**	**2.18**	**1.98; 2.41**	<0.001**	**2.78**	**2.58; 2.98**	<0.001**
**Afraid of partner**															
Never															
Most of the time	**7.87**	**6.17; 10.04**	<0.001**	**3.97**	**2.95; 5.34**	<0.001**	**14.38**	**9.28; 22.29**	<0.001**	**7.20**	**6.32; 8.21**	<0.001**	**6.78**	**6.14; 7.49**	<0.001**
Sometimes	**1.92**	**1.49; 2.46**	<0.001**	1.16	0.90; 1.48	0.250	**2.32**	**1.52; 3.54**	<0.001**	**2.57**	**2.26; 2.94**	<0.001**	**1.95**	**1.76; 2.16**	<0.001**
Community/societal characteristics															
**Place of residence**															
Urban															
Rural	**1.23**	**1.02; 1.48**	0.033*	**0.70**	**0.55; 0.89**	0.004*	**1.53**	**1.07; 2.19**	0.021*	**1.37**	**1.07; 1.77**	0.014*	**1.17**	**1.01; 1.36**	0.041*
**Wealth index**															
Poorer or Poorest															
Middle	0.97	0.68; 1.38	0.860	0.98	0.75; 1.29	0.891	1.02	0.67; 1.55	0.923	**0.84**	**0.74; 0.95**	0.005*	**0.88**	**0.80; 0.96**	0.004*
Richer or Richest	**1.60**	**1.08; 2.38**	0.019*	**0.70**	**0.52; 0.94**	0.016*	1.26	0.80; 1.98	0.314	**0.75**	**0.66; 0.86**	<0.001**	1.15	0.92; 1.44	0.225
**Decision making**															
Woman alone															
Woman and partner	**0.80**	**0.69; 0.92**	0.003*	**0.70**	**0.52; 0.94**	0.017*	0.80	0.49; 1.31	0.373	**0.73**	**0.62; 0.85**	<0.001**	**0.75**	**0.68; 0.84**	<0.001**
Partner alone	**1.54**	**1.23; 1.93**	<0.001**	**0.48**	**0.23; 1.00**	0.049*	0.88	0.50; 1.56	0.671	0.90	0.76; 1.05	0.180	**1.14**	**1.01; 1.29**	0.028*
**Controlling behavior: Partner...**															
jealous if respondent talks with other men/women	**4.23**	**3.63; 4.94**	<0.001**	**3.12**	**2.44; 3.98**	<0.001**	**2.42**	**1.64; 3.56**	<0.001**	**2.86**	**2.56; 3.20**	<0.001**	**3.50**	**3.22; 3.80**	<0.001**
accuses respondent of unfaithfulness	**4.00**	**3.47; 4.63**	<0.001**	**3.51**	**2.81; 4.39**	<0.001**	**3.95**	**2.60; 6.00**	<0.001**	**2.97**	**2.69; 3.28**	<0.001**	**3.47**	**3.22; 3.74**	<0.001**
does not permit respondent to meet female/male friends	**2.62**	**2.28; 3.01**	<0.001**	**3.54**	**2.83; 4.42**	<0.001**	**3.17**	**2.16; 4.65**	<0.001**	**2.84**	**2.57; 3.13**	<0.001**	**2.86**	**2.66; 3.08**	<0.001**
tries to limit respondent’s contact with family	**3.35**	**2.89; 3.87**	<0.001**	**3.26**	**2.57; 4.12**	<0.001**	**4.10**	**2.86; 5.89**	<0.001**	**2.97**	**2.66; 3.30**	<0.001**	**3.19**	**2.94; 3.45**	<0.001**
insists on knowing where respondent is	**3.34**	**2.92; 3.83**	<0.001**	**2.75**	**2.18; 3.46**	<0.001**	**2.52**	**1.85; 3.45**	<0.001**	**2.54**	**2.30; 2.81**	<0.001**	**2.92**	**2.71; 3.14**	<0.001**
**Wife beating justified if wife**															
goes out without telling husband	0.93	0.77; 1.13	0.470	0.84	0.46; 1.50	0.550	0.98	0.56; 1.71	0.951	0.94	0.81; 1.09	0.395	0.95	0.85; 1.06	0.363
neglects the children	0.82	0.63; 1.07	0.150	0.81	0.53; 1.22	0.313	0.64	0.37; 1.09	0.099	0.89	0.77; 1.02	0.103	0.98	0.93; 1.04	0.577
argues with husband	0.78	0.60; 1.03	0.082	0.74	0.34; 1.60	0.443	**0.52**	**0.29; 0.94**	0.031*	0.94	0.81; 1.09	0.434	0.96	0.90; 1.03	0.275
refuses to have sex with husband	0.89	0.65; 1.21	0.454	0.65	0.36; 1.20	0.170	**0.53**	**0.28; 0.99**	0.045*	**0.85**	**0.73; 0.99**	0.035*	1.03	0.96; 1.11	0.404
burns the food	0.77	0.57; 1.03	0.083	0.59	0.28; 1.27	0.180	**0.33**	**0.16; 0.71**	0.005*	0.93	0.78; 1.11	0.404	0.94	0.87; 1.02	0.128
**Health-seeking barriers (Following is a “big problem”)**															
getting permission to go	**2.48**	**2.04; 3.00**	<0.001**	**2.14**	**1.43; 3.18**	<0.001**	**1.74**	**1.11; 2.73**	0.016*	**1.20**	**1.02; 1.42**	0.029*	**2.50**	**2.14; 2.92**	<0.001**
getting money needed for treatment	**2.37**	**1.90; 2.96**	<0.001**	**3.28**	**1.90; 5.65**	<0.001**	1.28	0.88; 1.86	0.204	**1.25**	**1.12; 1.39**	<0.001**	**2.78**	**2.28; 3.39**	<0.001**
distance to health care facility	**2.36**	**1.81; 3.06**	<0.001**	**1.81**	**1.18; 2.77**	0.006*	**1.57**	**1.10; 2.24**	0.013*	0.99	0.89; 1.09	0.794	**2.42**	**1.96; 2.99**	<0.001**
not wanting to go alone	**2.43**	**1.97; 3.01**	<0.001**	**2.42**	**1.63; 3.60**	<0.001**	**1.94**	**1.40; 2.70**	<0.001**	0.99	0.88; 1.11	0.849	**2.60**	**2.18; 3.10**	<0.001**
**Sample size**		**2,399**			**1,231**			**553**			**3,513**			**7,696**	

aOR, adjusted Odds Ratio. 95% CI, 95% Confidence Interval. All results are weighted. ^**a**^ For Peru, only unweighted data was available, hence Peru’s data is excluded from regional and total weighted estimates. * p-value< 0.05. ** p-value< 0.001.

ORs were adjusted for women’s age, women’s age at first cohabitation, women’s education, women’s working status, women’s marital status, number of living children, partner’s education and working status, whether her partner drank alcohol, whether her father abused her mother, woman afraid of partner, wealth index and rural/urban residence

**Table 4:** Logistic Regression Odds Ratios (OR) of formal/institutional help-seeking for women experiencing IPV during pregnancy, by region, adjusted and weighted

	**Asia and Melanesia**			Latin America and Caribbean^a^			North Africa/ West Asia/Eastern Europe			Sub-Saharan Africa			Total^a^		
	aOR	95% CI	*p*-value	aOR	95% CI	*p*-value	aOR	95% CI	*p*-value	aOR	95% CI	*p*-value	aOR	95% CI	*p*-value
***Women’s characteristics***															
**Woman’s age (years)**															
15-19															
20-24	0.60	0.22; 1.62	0.314	**3.05**	**1.35; 6.87**	0.007*	1.06	0.46; 2.43	0.893	1.26	0.74; 2.16	0.394	1.41	0.90; 2.22	0.138
25-29	0.61	0.23; 1.63	0.326	**3.26**	**1.47; 7.22**	0.004*	1.17	0.52; 2.64	0.707	1.36	0.80; 2.31	0.256	1.38	0.89; 2.16	0.152
30-34	0.75	0.28; 2.01	0.563	**2.90**	**1.29; 6.56**	0.010*	1.19	0.52; 2.69	0.682	1.69	0.99; 2.89	0.053	1.54	0.99; 2.41	0.058
35-39	0.90	0.33; 2.47	0.836	**3.71**	**1.62; 8.51**	0.002*	1.16	0.51; 2.66	0.719	**1.75**	**1.02; 3.00**	0.043*	**1.75**	**1.11; 2.75**	0.017*
40-44	0.79	0.27; 2.26	0.654	**3.47**	**1.46; 8.21**	0.005*	1.04	0.45; 2.38	0.930	**1.85**	**1.06; 3.21**	0.030*	1.52	0.95; 2.41	0.079
45-49	0.90	0.25; 3.31	0.878	**4.14**	**1.74; 9.87**	0.001*	1.10	0.48; 2.52	0.824	**2.00**	**1.15; 3.47**	0.014*	**1.77**	**1.07; 2.93**	0.025*
**Age at first cohabitation (years)**															
<15															
15-19	1.17	0.82; 1.66	0.398	**1.61**	**1.17; 2.22**	0.004*	1.02	0.90; 1.15	0.789	1.05	0.87; 1.27	0.630	1.14	0.98; 1.32	0.097
20-24	0.77	0.52; 1.13	0.177	**0.61**	**0.41; 0.90**	0.013*	0.08	0.01; 1.03	0.053	**1.45**	**1.06; 1.97**	0.020*	0.89	0.75; 1.06	0.192
25+	**1.83**	**1.07; 3.13**	0.028*	**0.54**	**0.30; 0.96**	0.035*	0.99	0.83; 1.19	0.954	**0.55**	**0.34; 0.91**	0.019*	**1.19**	**1.00; 1.41**	0.048*
**Marital status**															
Previously married															
Currently married	**0.12**	**0.07; 0.20**	<0.001**	**0.34**	**0.24; 0.49**	<0.001**	**0.01**	**0.01; 0.05**	<0.001**	**0.28**	**0.23; 0.35**	<0.001**	**0.21**	**0.18; 0.25**	<0.001**
**Woman’s education**															
No education															
Primary	**2.83**	**1.80; 4.45**	<0.001**	**1.98**	**1.10; 3.57**	0.022*	**-**	**-**	-	**1.39**	**1.12; 1.73**	0.003*	**2.01**	**1.66; 2.44**	<0.001**
Secondary or higher	**3.98**	**2.52; 6.29**	<0.001**	**2.89**	**1.46; 5.72**	0.002*	1.01	0.77; 1.34	0.925	1.14	0.85; 1.52	0.377	**2.09**	**1.66; 2.64**	<0.001**
**Woman is working**															
No															
Yes	1.55	0.99; 2.42	0.053	1.30	0.93; 1.83	0.126	0.98	0.86; 1.11	0.728	**1.56**	**1.23; 1.98**	<0.001**	**1.99**	**1.68; 2.35**	<0.001**
**Who earns more**															
About the same															
Husband/partner earns more	0.75	0.31; 1.84	0.530	1.13	0.53; 2.42	0.747	1.03	0.78; 1.35	0.846	1.00	0.77; 1.30	0.988	**1.72**	**1.19; 2.50**	0.004*
Woman earns more than him	**2.86**	**1.21; 6.76**	0.017*	0.84	0.30; 2.36	0.738	1.02	0.65; 1.60	0.926	**1.77**	**1.25; 2.51**	0.001*	**1.81**	**1.15; 2.84**	0.010*
Exposure to mass media (at least once a week)															
Reading newspaper or magazine	**2.23**	**1.19; 4.16**	0.012*	1.27	0.95; 1.71	0.108	1.01	0.86; 1.18	0.914	**1.49**	**1.04; 2.15**	0.032*	**1.34**	**1.04; 1.72**	0.024*
Listening to radio	1.00	0.68; 1.48	0.987	0.87	0.60; 1.25	0.443	0.91	0.74; 1.11	0.364	**1.25**	**1.02; 1.53**	0.031*	**1.44**	**1.23; 1.68**	<0.001**
Watching television	**0.44**	**0.26; 0.73**	0.002*	**1.68**	**1.15; 2.44**	0.007*	1.03	0.52; 2.05	0.932	0.77	0.57; 1.04	0.093	**0.61**	**0.50; 0.75**	<0.001**
**Current pregnancy wanted**															
Later															
Then	0.66	0.14; 3.12	0.604	1.08	0.16; 7.39	0.934	0.67	0.03; 14.98	0.794	0.57	0.32; 1.04	0.066	**0.58**	**0.35; 0.97**	0.040*
Not at all	0.15	0.02; 1.42	0.098	0.59	0.05; 6.77	0.666	0.51	0.03; 7.68	0.612	0.74	0.31; 1.78	0.503	0.82	0.39; 1.76	0.618
***Partner/family characteristics***															
**Partner’s age (years)**															
<21															
21-30	0.79	0.45; 1.36	0.392	1.10	0.66; 1.85	0.712	0.93	0.76; 1.13	0.455	1.05	0.78; 1.43	0.737	1.06	0.83; 1.36	0.630
31-40	1.17	0.74; 1.85	0.512	1.14	0.77; 1.71	0.509	1.05	0.92; 1.20	0.438	1.14	0.90; 1.44	0.274	1.10	0.91; 1.33	0.347
41-50	0.96	0.61; 1.51	0.869	0.71	0.44; 1.14	0.153	0.97	0.84; 1.12	0.690	**0.71**	**0.51; 0.99**	0.040*	**0.76**	**0.59; 0.97**	0.031*
51-60	0.42	0.16; 1.10	0.077	0.78	0.41; 1.48	0.443	1.01	0.81; 1.25	0.948	**1.60**	**1.02; 2.51**	0.042*	1.12	0.84; 1.49	0.448
>60	0.42	0.10; 1.80	0.242	0.18	0.02; 2.06	0.170	1.21	0.47; 3.12	0.690	0.77	0.39; 1.52	0.458	0.88	0.49; 1.56	0.658
**Partner’s education**															
No education															
Primary	**1.56**	**1.02; 2.36**	0.038*	1.09	0.65; 1.84	0.736	0.81	0.20; 3.33	0.775	**1.55**	**1.18; 2.04**	0.002*	**1.58**	**1.25; 1.99**	<0.001**
Secondary or higher	1.20	0.74; 1.95	0.451	1.21	0.59; 2.50	0.598	1.10	0.57; 2.12	0.778	0.94	0.66; 1.35	0.755	1.10	0.84; 1.44	0.489
**Partner is working**															
No															
Yes	**0.29**	**0.18; 0.46**	<0.001**	1.18	0.26; 5.46	0.831	1.04	0.84; 1.30	0.692	**0.46**	**0.26; 0.80**	0.006*	**0.55**	**0.38; 0.80**	0.002*
**Partner drinks alcohol**															
Never															
Often	**3.17**	**1.62; 6.22**	<0.001**	**1.79**	**1.13; 2.83**	0.013*	1.12	0.52; 2.39	0.769	**7.01**	**3.95; 12.44**	<0.001**	**5.73**	**3.58; 9.17**	<0.001**
Sometimes	1.31	0.69; 2.49	0.410	1.02	0.66; 1.55	0.943	1.21	0.59; 2.47	0.597	**2.49**	**1.39; 4.47**	0.002*	**1.61**	**1.01; 2.57**	0.047*
**Number of living children**	**1.13**	**1.04; 1.23**	0.003*	**1.14**	**1.03; 1.26**	0.009*	**1.96**	**1.42; 2.71**	<0.001**	**1.10**	**1.05; 1.16**	<0.001**	**1.16**	**1.12; 1.20**	<0.001**
Women’s father abused her mother															
No															
Yes	**3.55**	**2.45; 5.16**	<0.001**	**2.04**	**1.49; 2.80**	<0.001**	1.01	0.80; 1.28	0.923	**2.24**	**1.87; 2.70**	<0.001**	**2.46**	**2.12; 2.85**	<0.001**
**Afraid of partner**															
Never															
Most of the time	**6.83**	**4.22; 11.04**	<0.001**	**2.63**	**1.71; 4.04**	<0.001**	1.04	0.57; 1.87	0.906	**7.93**	**6.15; 10.21**	<0.001**	**5.32**	**4.40; 6.44**	<0.001**
Sometimes	**1.79**	**1.01; 3.17**	0.045*	0.81	0.56; 1.18	0.274	0.93	0.64; 1.35	0.687	**2.12**	**1.64; 2.75**	<0.001**	**1.46**	**1.19; 1.79**	<0.001**
***Community/societal characteristics***															
**Place of residence**															
Urban															
Rural	0.91	0.59; 1.41	0.667	**0.50**	**0.33; 0.76**	0.001*	0.95	0.81; 1.11	0.507	1.11	0.90; 1.38	0.338	**0.84**	**0.73; 0.97**	0.014*
**Wealth index**															
Poorer or Poorest															
Middle	0.66	0.43; 1.02	0.064	1.01	0.67; 1.52	0.960	**0.09**	**0.01; 0.84**	0.035*	**1.28**	**1.00; 1.64**	0.049*	1.23	0.95; 1.59	0.120
Richer or Richest	**1.65**	**1.21; 2.24**	0.001*	**0.48**	**0.30; 0.77**	0.003*	0.33	0.04; 3.10	0.332	**1.73**	**1.36; 2.22**	<0.001**	**1.39**	**1.05; 1.84**	0.022*
**Decision making**															
Woman alone															
Woman and partner	0.78	0.48; 1.27	0.325	0.89	0.66; 1.20	0.439	1.01	0.59; 1.72	0.971	**0.73**	**0.55; 0.97**	0.031*	**0.71**	**0.61; 0.83**	<0.001**
Partner alone	**0.50**	**0.26; 0.97**	0.040*	**0.53**	**0.32; 0.86**	0.010*	0.80	0.31; 2.09	0.651	**0.69**	**0.50; 0.94**	0.019*	**0.67**	**0.56; 0.79**	<0.001**
**Controlling behavior: Partner...**															
jealous if respondent talks with other men/women	**2.03**	**1.34; 3.08**	<0.001**	**2.70**	**1.87; 3.92**	<0.001**	1.01	0.89; 1.15	0.901	**3.02**	**2.44; 3.75**	<0.001**	**3.54**	**2.98; 4.19**	<0.001**
accuses respondent of unfaithfulness	**2.70**	**1.91; 3.82**	<0.001**	**3.68**	**2.69; 5.03**	<0.001**	1.10	0.60; 2.00	0.764	**2.97**	**2.44; 3.60**	<0.001**	**4.30**	**3.68; 5.02**	<0.001**
does not permit respondent to meet female/male friends	**1.45**	**1.01; 2.08**	0.046*	**2.67**	**1.94; 3.69**	<0.001**	0.93	0.72; 1.21	0.594	**3.01**	**2.49; 3.64**	<0.001**	**3.21**	**2.76; 3.74**	<0.001**
tries to limit respondent’s contact with family	**1.59**	**1.10; 2.29**	0.014*	**2.52**	**1.78; 3.56**	<0.001**	0.94	0.62; 1.41	0.755	**4.08**	**3.35; 4.98**	<0.001**	**4.04**	**3.46; 4.73**	<0.001**
insists on knowing where respondent is	**1.83**	**1.30; 2.56**	<0.001**	**2.25**	**1.61; 3.14**	<0.001**	0.98	0.86; 1.11	0.722	**2.74**	**2.25; 3.35**	<0.001**	**3.26**	**2.80; 3.79**	<0.001**
**Wife beating justified if wife**															
goes out without telling husband	0.75	0.48; 1.15	0.188	**0.14**	**0.04; 0.50**	0.002*	1.08	0.85; 1.37	0.534	**0.87**	**0.76; 0.99**	0.033*	**0.76**	**0.64; 0.91**	0.002*
neglects the children	1.15	0.76; 1.72	0.507	**0.43**	**0.20; 0.92**	0.029*	0.99	0.58; 1.67	0.965	**0.74**	**0.57; 0.96**	0.025*	**0.78**	**0.64; 0.96**	0.019*
argues with husband	**0.63**	**0.40; 0.98**	0.043*	**0.16**	**0.04; 0.68**	0.012*	1.09	0.87; 1.36	0.472	**0.75**	**0.61; 0.93**	0.007*	**0.68**	**0.57; 0.81**	<0.001**
refuses to have sex with husband	1.08	0.67; 1.72	0.754	0.47	0.20; 1.12	0.090	0.87	0.42; 1.79	0.700	0.84	0.68; 1.04	0.103	0.92	0.77; 1.10	0.362
burns the food	0.96	0.60; 1.53	0.859	0.45	0.16; 1.24	0.121	1.22	0.41; 3.62	0.723	0.81	0.63; 1.05	0.112	0.89	0.73; 1.09	0.255
**Health-seeking barriers (Following is a “big problem”)**															
getting permission to go	**1.87**	**1.12; 3.13**	0.016*	0.91	0.49; 1.67	0.754	0.35	0.05; 2.56	0.301	**0.71**	**0.53; 0.94**	0.018*	**2.38**	**1.67; 3.40**	<0.001**
getting money needed for treatment	**2.66**	**1.59; 4.44**	<0.001**	**2.22**	**1.29; 3.82**	0.004*	**0.07**	**0.01; 0.45**	0.005*	**1.29**	**1.06; 1.56**	0.010*	**3.71**	**2.61; 5.28**	<0.001**
distance to health care facility	**2.66**	**1.42; 4.97**	0.002*	0.93	0.47; 1.86	0.842	0.49	0.10; 2.53	0.396	**0.83**	**0.73; 0.95**	0.005*	**1.99**	**1.34; 2.97**	<0.001**
not wanting to go alone	**2.67**	**1.46; 4.87**	0.001*	**1.61**	**1.07; 2.42**	0.023*	0.20	0.03; 1.32	0.095	1.05	0.84; 1.33	0.657	**2.31**	**1.57; 3.40**	<0.001**
**Sample size**		**305**			**255**			**125**			**916**			**1,600**	

aOR, adjusted Odds Ratio. 95% CI, 95% Confidence Interval. All results are weighted. ^**a**^ For Peru, only unweighted data was available, hence Peru’s data is excluded from regional and total weighted estimates. * p-value< 0.05. ** p-value< 0.001. **–** Data not available.

ORs were adjusted for women’s age, women’s age at first cohabitation, women’s education, women’s working status, women’s marital status, number of living children, partner’s education and working status, whether her partner drank alcohol, whether her father abused her mother, woman afraid of partner, wealth index and rural/urban residence

**Table 5:** Descriptive statistics of reasons for not seeking help among women experiencing IPV during pregnancy, by country, weighted

	Dominican Republic			Peru^a^			Burkina Faso			Ivory Coast			Total		
	Did not seek help (%)	*p*-value	chi-square	Did not seek help (%)	*p*-value	chi-square	Did not seek help (%)	*p*-value	chi-square	Did not seek help (%)	*p*-value	chi-square	Did not seek help (%)	*p*-value	chi-square
**Main reason never sought help**		<0.001**	**18.232**		0.001**	**25.136**		<0.001**	**4.1617**		0.003**	**3.3738**		<0.001**	**3.8624**
Don’t know where to go to	9.80			18.95			17.20			14.65			36.31		
It was of no use	24.63			5.26			32.72			37.12			28.53		
It is part of life	6.17			2.11			31.64			12.71			15.68		
Afraid of divorce	11.03			5.26			2.58			9.15			4.35		
Afraid of further beatings	22.55			21.32			3.22			8.04			4.12		
Afraid of getting the person in trouble	13.67			10.79			2.72			9.52			4.54		
Embarrassed	12.14			22.63			6.65			6.77			4.68		
Don’t want to disgrace family	0.00			-			3.27			2.03			1.79		
It was her (woman’s) fault	-			0.26			-			-			-		
It was not necessary	-			13.42			-			-			-		

All variables are expressed as proportions (in %). All results are weighted. Chi-square and p-value concern the non-help-seeking of IPV-exposed women during pregnancy.

^**a**^ For Peru, only unweighted data was available, hence Peru’s data is excluded from total weighted estimates. ** p-value< 0.005

## Discussion

The global prevalence of experiencing IPV during pregnancy of 5.63% falls within the ranges reported in previous multi-country studies [2, 10–12]. Although we revealed that only half of women (51.87%) sought help for IPV during pregnancy - regardless of source - this rate was higher than reported by previous cross-national studies [2, 11, 25–27]. Consistent with prior research [2, 11, 25–27, 29–31, 33, 38, 41, 47], we found that IPV survivors seek mostly informal help from family, followed by in-laws, neighbors, and friends, rather than from formal institutions, such as police, followed by social service organizations, lawyers, religious leaders, and lastly medical professionals. For informal help-seeking, the lowest prevalence came from Latin America and Caribbean, and the highest from sub-Saharan Africa, whereas for formal help-seeking, the lowest rates were detected in Asia and Melanesia, and the highest in Latin America and Caribbean, of which the latter was also described elsewhere [2, 26]. Compared to other regions, survivors sought generally much less help for IPV in Asia and Melanesia, where the lowest regional rates were detected for any and formal help-seeking, the second lowest rates for informal help-seeking and the greatest difference between informal and formal help-seeking percentages (36.65% vs. 4.66%, respectively). Interestingly, in Latin America and Caribbean, the lowest and the highest regional help-seeking prevalences were found (informal and formal, respectively), but at the same time the difference between the help-seeking rates from informal and formal sources was the smallest (35.12% vs. 16.21%, respectively). In North Africa/West Asia/Eastern Europe, regional prevalences for any, informal, and formal help-seeking were ascertained to be continuously the second highest, with the lowest regional percentage of IPV during pregnancy (4.08%). In sub-Saharan Africa, the highest regional rates for any and informal help-seeking were determined, but simultaneously the largest prevalence differences were found on a national level (each <0.01% in Senegal vs. 86.27% and 80.24% in Liberia for any and informal help, respectively). On a national level, the lowest help-seeking rates were identified continuously in Senegal (<0.01%) and frequently in Armenia (<0.01% for help-seeking from friends and all formal sources). Sierra Leone was ascertained as the country with the highest percentages of informal help-seeking from in-laws (42.93%) and friends (23.83%), and Moldova as the country with the highest rates of formal help-seeking from police and medical professionals (27.16% and 5.78%, respectively).

The low overall help-seeking rates may be explained by an interplay of survivors’ individual agency, beliefs, and social context, as well as individual, interpersonal, and sociocultural causes and stigmata which have implications for how survivors define IPV, and whether and from whom they are willing to seek help [3, 21, 48].

On women’s individual level, stigma internalization including shame, embarrassment, guilt, self-blame, self-doubt, low self-esteem, low self-worth, and low self-efficacy can impede women from recognizing IPV as an intolerable problem for which help should be sought [21, 48]. This should not be understood as victim-blaming for not seeking help, but instead should offer some explanations for the internal psychological processes possibly taking place in IPV survivors which might be related with no help-seeking. Furthermore, women who think they can stop the violence themselves, who deny and trivialize abuse, who want to protect the abusive partner - as well as those who are timid, reticent, uncommunicative, or isolated - might be less likely to seek help from others [3].

On an interpersonal level, anticipated stigma, such as concerns about other people’s prejudice, discrimination, rejection, disapproval, devaluation, and the fear of being judged or ridiculed or losing the job, can hinder IPV survivors to seek help [48]. It can also be challenging (and confounding) to define a relationship as violent, as abuse in an intimate partnership might be permanently changing, with different severity, occurrence, and patterns, and perpetrators varying from abuse to remorse and loving behavior [3].

On a sociocultural level, cultural stigmata can be barriers to help-seeking, including victim-blaming, judgmental attitudes, and the perception of abuse as a personal issue or private matter or a normal incident [48]. IPV survivors’ gender role expectations and cultural identifications - such as being obedient, subordinating, self-sacrificing, and responsible to keep the family unified - can prevent them from seeking help [3].

The low rates of help-seeking from formal institutions might be elucidated by manifold material, financial, language, structural, logistical, and politico-legal barriers [3, 5, 11, 31, 32, 38, 49]. In particular, lack of trust in the system and institutions, perceived lack of access to help, low knowledge about rights and resources, lack of money, health insurance and time, as well as child care or transportation issues can be barriers to seek help from formal sources [5, 31]. Furthermore, negative perceptions of institutional support services as not needed, not useful or effective, discriminatory, racist, and inaccessible - especially for migrants, older women, and drug users - can inhibit women from seeking help for IPV [5, 11, 21, 31]. In addition, routine IPV screening is not always available within the standard maternal health care offerings in LMICs [23]. This is particularly problematic because in resource-limited settings, women often access healthcare for the first time when they are pregnant, and an in-depth and holistic healthcare provision for women might often be overlooked [23].

Our study has shown that seeking help for IPV during pregnancy is significantly associated with several facilitating and inhibiting factors. Among women’s individual characteristics, older age (35-49 years) was positively associated with formal help-seeking, which is consistent with previous findings [26, 27, 50]. This might be explained by women’s increased independence from their partners and higher social status with more self-determination and autonomy, which might arise with increasing age. Additionally, older women might have experienced abuse over a longer period of time and not seek help until the extent, severity and frequency of violence were no longer acceptable [5, 27, 29, 51–54]. Furthermore, the process of help-seeking can be tremendously challenging and complex, hence it might take many years for IPV survivors to decide to proceed against their abusers and to seek help from legal or other formal institutions [51].

In spite of regional variations, women who cohabitate for the first time at older ages are more likely to seek help in response to IPV during pregnancy, which is consistent with previous studies [27]. Researchers state that an older age at first marriage/cohabitation might be related to more educational attainment, more decision in choosing a husband, more time to gain a strong self-awareness outside the marriage, and less risk for intimidation and acceptance of violent behavior; consequently, leading to more rejection of IPV [34, 55].

Married women are less likely to seek help, which is an often-cited phenomenon in the existing literature [26, 29, 52, 56, 57]. Barriers for married women to end their violent partnership might be love for the partner, willingness to protect him, cultural and religious beliefs around the preservation of marriage and the family’s reputation, as well as fear of social stigmatization regarding divorce, combined with feelings of shame, embarrassment, and guilt [5, 7, 11, 31, 32, 57]. This is consistent with our findings that “being afraid of divorce”, “being afraid of getting the person in trouble”, “being embarrassed”, and “don’t want to disgrace family” were main reasons for IPV survivors to never seek help (Table 5, Descriptive statistics of reasons for not seeking help among women experiencing IPV during pregnancy, by country, weighted). In addition, in some cultures (e.g., in Kyrgyzstan), when a woman is married, she is no longer under the protection of her natal family, thus, it may deter her from seeking help from her own relatives [3, 57].

Primary, and secondary or higher education of both, women and their partners, were associated with higher odds of help-seeking for IPV during pregnancy, which was similarly presented in prior studies [5, 27, 30, 35]. Higher educational attainment may widen women’s and men’s horizons increasing their access to a broader range of conflict resolution skills and a rejection of IPV, while, at the same time, broadening IPV survivors’ knowledge about their rights and resources to seek help [34, 55, 58]. In turn, more literate women may also obtain more opportunities to be employed and fend for themselves in case of leaving their abusive partners [34].

In line with the latter and previous findings [27, 30, 35, 50, 52, 56, 59], women who were employed and earned more money than their husbands were more likely to seek help. This phenomenon might be elucidated by the theories of economic and marital dependencies, which state that employed women are more economically independent from their partners, and, therefore, are more capable to end an abusive relation [6, 35, 51]. Economic and marital dependence might aggravate particularly for pregnant women because they often cannot work, especially at later stages of pregnancy. In addition, paid maternity leave, state social assistance and unemployment benefit might be limited in some LMICs. Moreover, working women have more social contacts and opportunities outside marriage [58], which might encourage them to seek help and simultaneously to imagine and achieve a life without their abuser. At the same time, having a husband who was working and earned more money than her also encouraged IPV survivors to seek help. This might be a sign of unequal roles within a marriage and of behavioral patterns that battered women might seek to escape from.

Exposure to mass media (at least once a week), including newspaper or magazine and radio, was positively associated with help-seeking, whereas watching television was negatively associated with help-seeking. This might partly be reflected in women’s increased access to an intolerant mindset against IPV and to more gender egalitarian ideas through media [55, 60]. Print and radio consumption might be aimed more at obtaining information, while consuming television might be more for entertaining purposes, which might partly explain the different help-seeking behaviors associated with exposure to different media sources. Additionally, access to high-quality media might entail more information about where to seek help (e.g., helplines or online platforms of social services or non-government organizations) [61]. This is also consistent with our finding that “don’t know where to go to” was a main reason for IPV survivors to never seek help (Table 5, Descriptive statistics of reasons for not seeking help among women experiencing IPV during pregnancy, by country, weighted).

Whereas partner’s younger age of 21-40 years was associated with higher odds of women’s help-seeking, partner’s older age of 41 years and above was associated with lower odds, which might be elucidated by the change of abuse patterns with regard on men’s age. First, younger men might be physically stronger and healthier than older men, and therefore might threaten or harm women more severely and frequently. Consequently, women might seek more help. Second, women having older partners might seek less help because IPV generally diminishes as the husbands’ age increases [62]. Third, women’s cultural beliefs such as “respect for elders, caregiving as a dominant role and commitment to the institution of marriage” might hinder them from help-seeking [62].

Consistent with existing findings [27, 29, 30], the more often the partner consumed alcohol and the more often a woman was afraid of her partner, the higher was the likelihood for her to seek help in response to IPV during pregnancy. It is well-known in empirical research that partner’s alcohol consumption is associated with a higher risk, severity, and frequency of perpetrating IPV during pregnancy and with causing heightened fear in women [36, 63–65], which, in turn, leads to more help-seeking [54]. Furthermore, pregnancy can be a challenging stage in life for becoming parents with increased stress, affiliated with social role transitions, physical, emotional, social and economic burdens, as well as pandemic-related difficulties during COVID-19 [4, 8, 9, 66]. Thus, men, in particular those with low psychological flexibility and maladaptive cognitive emotion regulation strategies, might cope with these stressors by increasing their alcohol abuse which results in more perpetration of IPV [67–69] and, subsequently, in more help-seeking.

An increasing number of living children facilitated women’s help-seeking for IPV during pregnancy, which might be explained by the mothers’ fear that their children might be hurt or menaced by the abusive partner, or might witness violence [5]. These fears might particularly be a pregnant woman’s concern who wants to protect her unborn child and ensure that it will be born in a safe environment. However, this stands in contrast to some research findings stating that having children could also discourage women to seek help due to their fear of losing them, and their desire for their children to grow up with an intact family, home and financial stability [5, 32, 51, 57].

Pregnant women who had witnessed their fathers abusing their mothers were more likely to seek informal and formal help in response to IPV, as prior research has also demonstrated [27, 56]. Given that we could not investigate informal support networks separately, it may be that IPV survivors who had witnessed violence perpetrated by their fathers against their mothers seek more help from their neighbors, friends, in-laws, and formal institutions, because support from their own families is rather limited [70]. Moreover, realizing that witnessing parental violence entails a higher risk for violence experienced by oneself later in life [4], pregnant women might want to break the cycle of ongoing violence not only for themselves but also for the child they are carrying, and therefore might be more likely to seek help.

Consistent with previous studies [26, 30, 50, 71], IPV survivors living in a rural area were more likely to seek informal help but, simultaneously, were less likely to reach out for formal help. In rural settings, the access to healthcare facilities, police stations, lawyers, and social services is often limited, and women’s trust in these institutions is lacking [5, 71]. Consequently, pregnant women might have no other option than to reach out to family members, neighbors, and friends. It is also easy to imagine that traveling long distances might be a high burden for pregnant women, particularly at later pregnancy stages, besides other material, financial and logistical barriers to reach formal services [5, 9, 31, 38]. Additionally, there is evidence that among women living in rural areas, IPV is more widely accepted and, therefore, justified [55, 60]. Thus, they might be less likely to take legal proceedings against their abusers.

Despite regional variations, the pooled odds of help-seeking in response to IPV during pregnancy decreased when having a middle wealth index and increased when belonging to the richer or richest wealth quintile, which is in line with findings from other researchers [29, 30, 50, 56]. These results are consistent with economic and marital dependency theories which suggest that women who are financially dependent on their partners are less able to end an abusive relationship [6, 35], going hand in hand with women’s employment status and salary differences between partners, as aforementioned. Since pregnancy and the future child imply an increased financial burden, pregnant women might be especially vulnerable when they are poor or depend economically on their husbands, and therefore condone IPV [7]. In contrast, in those regions where wealthier women had lower odds of help-seeking, they might be afraid that their disclosure of violence would endanger their economic prestige and status [35].

If decisions were made jointly, women were predominantly less likely to seek any form of help for IPV, in comparison with woman alone as the final decision makers, which coincides with previous findings [27, 72]. Similarly, having a partner alone as the decision maker hindered survivors’ formal help-seeking. It is well conceivable that a woman who has the final say in decisions concerning healthcare, household purchases, spending, or visiting relatives and friends, may have at the same time more autonomy in the decision to move and think freely and to seek help. Additionally, women’s increased decision-making capacity within a partnership may reflect their beliefs in gender equality, emancipation and their own empowerment, and thus foster their rejection of IPV [60, 72].

Controlling behaviors exercised by a partner facilitated IPV survivor’s help-seeking, as existing studies have also indicated [27, 30]. A husband’s jealousy, suspicion of unfaithfulness, and limitation of a wife’s contacts and freedom might raise her awareness that his behaviors are problematic and unacceptable [72], and on top of her experience of his violence might be the straw that breaks the camel’s back. This might be essential in the first stage of help-seeking, which is the problem recognition and definition [3]. Furthermore, men’s controlling behaviors might exacerbate during pregnancy due to uncertainty of male paternity, jealousy, and possessiveness [4], which are also additional drivers for violence against pregnant women [36], and consequently might encourage women to seek help.

It is easily comprehensible that women who believe that wife beating is justified (e.g., when going out without telling husband, arguing with him, refusing to have sex with him, neglecting the children, or burning the food), might not question their abuse and thus are less likely to seek help in response to IPV during pregnancy. This is consistent with our findings that some IPV survivors never sought help due to their beliefs that violence is “part of life” or “her own fault”, or that help “is not necessary” (Table 5, Descriptive statistics of reasons for not seeking help among women experiencing IPV during pregnancy, by country, weighted). In societies with social norms which allow or enable IPV, particularly in resource-poor settings, violence is often considered to be normal and legitimized [5, 7]. In line with this, empirical research has revealed that women, people of younger age, those with less education, poor wealth status, rural residence, and less access to media are more likely to accept IPV against women [55, 58, 60], of which the four latter circumstances were also found in this study to be associated with lower help-seeking for IPV. Moreover, Pierotti [55] states that the diffusion of global cultural scripts accounts for the increasing rejection of IPV worldwide, and that education, development programs, NGO work, and interpersonal networks play their part in this trend.

Consistent with previous research [33], facing “big problems” to access health care (including getting permission to go, getting money needed for treatment, distance to health care facility, or not wanting to go alone) generally fostered IPV survivors’ help-seeking, especially from informal support networks. Addressing the third stage of help-seeking (access to and selection of support) [3], barriers to accessing health care might also reflect a limited access to other institutional services. Consequently, IPV survivors facing these barriers are less likely to seek formal help (which was found to be the case in North Africa/West Asia/Eastern Europe and sub-Saharan Africa), and are forced to reach out to family, friends, and neighbors instead, particularly during pregnancy when their physical, emotional, and socioeconomic vulnerability is increased [4, 8]. During the COVID-19 pandemic access to support and health care services can be even more limited and problematic due to “stay-at-home” public health mandates and legal restrictions [14]. Further barriers to seek help for IPV can be language problems, healthcare or service centre’s professionals’ negative attitudes toward violence survivors, their lack of empathy, time and training, the fact that they simply do not ask about IPV, and no available female counsellor, as indicated in the existing literature [5, 32, 38, 42].

**Strengths and limitations** The strength of this study is that we made use of cross-sectional, population-based, and nationally representative data from the Demographic and Health Surveys, which enabled comparisons of help-seeking behaviors on a global scale. A limitation of the study is that the DHS datasets preclude any causal inference, and thus fail to fully understand in detail why women decide to seek help from a specific source and why not. Additional limitations of the data itself are the quantitative cross-sectional nature of the data, the sex and age range of respondents, as well as inconsistencies in variable wording and answer options, and partial unavailability of data. Our study was also limited by potential recall bias, as help-seeking estimates might be underreported due to women’s fear that participating in interviews could provoke further violence or social stigma. Moreover, because DHS data is only collected in households, it lacks crucial information about refugees, internally displaced persons, homeless people, and other vulnerable minorities. Another limitation is that we cannot fully ascertain whether respondents were reporting about help-seeking during pregnancy or another time, as the DHS survey question on help-seeking refers to all the types of violence women have experienced. Hence, the numbers of help-seeking during pregnancy could be overcounted. Finally, our analysis was limited by the lack of data in some countries and areas, particularly in humanitarian and conflict settings, and by the fact that some of the most recent datasets were more than a decade old.

**Future research**

Future research should focus on the specific barriers that pregnant women report when seeking help from informal networks and especially formal institutions, examining several levels at the same time (individual, partnership, community, and society). Another important aspect is the need for further research on how social media use influences help-seeking behaviors. Future research attention should also be paid to an in-depth understanding of the magnitude and nature of help-seeking in response to violence, conducting not only quantitative, but also qualitative studies shedding light on both, female and male perspectives of this issue.

## Conclusion

Whereas prior research attention has been given to women’s help-seeking behaviors on a country-specific level, in high-income countries, among non-pregnant women, and/or focusing on only one form of help-seeking, the present study provides timely cross-national evidence on the nature of help-seeking behaviors of pregnant women who are exposed to violence within intimate partnership contexts in LMICs. We revealed that 5.63% of women worldwide experienced IPV during pregnancy, and only half of them sought help to stop this violence, mainly from informal support networks, and rarely reached out to formal institutions, with wide variations between regions and countries.

The present study indicates that women’s, partner’s, and community’s factors facilitate and inhibit IPV survivors’ help-seeking similarly in developing nations, despite regional discrepancies. Hence, all these individual, familial, and societal determinants associated with help-seeking should be taken into account when policies, interventions, and networks aim to increase and improve opportunities for IPV survivors to seek support.

Our analysis has shown that particularly pregnant women who experience IPV face several physical, mental, educational, social, cultural, material, financial, and logistic barriers when seeking help; this must be understood in light of increased challenges, stress, and vulnerabilities which pregnancy itself involves for becoming parents, related to physical, emotional, social, and economic burdens [4, 8, 9].

To foster women’s help-seeking for IPV during pregnancy their marital and economic dependencies on their partners must be reduced by simultaneously strengthening their autonomy, decision-making capacity, and socioeconomic independence. Going hand in hand with the latter, reducing poverty, ensuring educational attainment, creating the economic conditions necessary for employment opportunities, and disseminating information about help sources, for example through mass media, are recommended interventions to improve help-seeking behaviors of pregnant women subjected to IPV.

The findings highlight the need for programmatic activities within communities which emphasize the diffusion of gender equality and the rejection and elimination of IPV by empowering the community as a whole, and engaging women and men equally. Efforts should be made on routine screening for IPV within health service provision for pre- and peri-natal care [23, 73], referral pathways, home visitation programs, supportive counselling interventions [23, 74], and similar therapeutic adjuncts involving the individuals in the intimate relationship.

To conclude, interventions and changes are needed, ranging in scope from the individual to familial and societal levels, to improve help-seeking in response to IPV during pregnancy, and to break the cycle of ongoing violence, not only for pregnant women, but also for their unborn children, their families, and societies at large.

## Supplementary information

**Additional file 1.** Supplementary Material 1.

**Additional file 2.** Supplementary Material 2.

## Data Availability

This study utilises data previously collected by the Demographic and Health Survey (DHS) Program from 54 low- and middle-income countries between 2005 and 2020 (the fifth to eight survey waves). The data used in this study are available in anonymised form (www.dhsprogram.com/data/available-datasets.cfm) upon request and with permission of ICF International, which authorised permission for using the data for this research. Additional information about the DHS program, questionnaires, procedures, and methods can be obtained from the official DHS website (www.dhsprogram.com).
