# Supplementary material for: Help-seeking behaviors among survivors of intimate partner violence during pregnancy in 54 low- and middle-income countries: evidence from Demographic and Health Survey data
Source: BMC Public Health. 2025 Feb 1;25:413. doi: 10.1186/s12889-025-21421-3 (PMC11787739; doi:10.1186/s12889-025-21421-3)
Supplement: Supplementary file 1 — Additional file 1. Supplementary Material 1. [file 12889_2025_21421_MOESM1_ESM.pdf]

Supplement: Table 1. Descriptive statistics among women experiencing IPV during pregnancy, by region, weighted.

	Asia and Melanesia				Latin America and Caribbean[a]				North Africa/West Asia/Eastern Europe			
	Sought help (%)	Did not seek help (%)	*p-value*	chi-square or t-value	Sought help (%)	Did not seek help (%)	*p-value*	chi-square or t-value	Sought help (%)	Did not seek help (%)	*p-value*	chi-square or t-value
Women's characteristics												
**Woman's age (years)**			0.205	1.4216			0.749	0.55901			0.120	1.6927
15-19	2.08	2.67			3.88	3.78			1.65	2.34		
20-24	12.91	15.05			11.31	12.09			12.06	14.10		
25-29	18.40	20.60			15.55	15.08			15.41	21.04		
30-34	20.06	16.88			17.53	19.57			19.53	11.30		
35-39	18.27	16.99			19.58	16.25			19.46	18.99		
40-44	12.77	13.23			16.65	16.63			15.65	16.98		
45-49	15.50	14.58			15.49	16.61			16.23	15.26		
**Age at first cohabitation (years)**			0.096	1.6642			0.561	0.58142			0.216	1.2372
<15	12.86	16.55			14.40	17.17			4.35	5.79		
15-19	55.29	52.14			56.88	54.73			49.83	50.72		
20-24	24.96	25.79			22.35	20.74			34.26	33.07		
25+	6.89	5.52			6.37	7.36			11.57	10.43		
**Marital status**			**<0.001****	**28.155**			0.416	0.66073			**0.004***	**8.3464**
Previously married	12.61	6.55			39.07	36.85			33.37	22.04		
Currently married	87.39	93.45			60.93	63.15			66.63	77.96		
**Woman's education**			**<0.001****	**8.2213**			0.101	2.3265			**0.027***	**3.6354**
No education	54.92	63.92			5.55	7.28			11.22	16.50		
Primary	16.84	14.35			39.04	41.96			6.96	11.07		
Secondary or higher	28.25	21.73			55.41	50.76			81.82	72.43		
**Woman is working**			0.075	3.1699			0.773	0.083386			0.092	2.4386
No	67.37	70.71			38.69	39.44			59.47	67.44		
Yes	32.63	29.29			61.31	60.56			40.53	32.56		
**Who earns more**			0.559	0.57448			0.104	2.2908			0.222	1.5152
About the same	16.69	16.14			20.47	17.82			14.75	28.20		
Husband/partner earns more	60.99	64.93			67.40	73.71			54.45	59.79		
Woman earns more than him	22.32	18.93			12.12	8.47			30.80	12.02		
**Exposure to mass media (at least once a week)**												
Reading newspaper or magazine	8.85	6.04	**0.008***	**4.2019**	29.67	26.91	0.663	0.41131	21.98	18.04	**0.025***	**3.7289**
Listening to radio	15.38	18.46	0.067	2.3975	66.56	62.52	0.416	0.87705	33.18	28.69	0.064	2.7486
Watching television	32.65	25.11	**<0.001****	**6.7333**	69.32	70.44	0.864	0.14574	86.78	81.16	0.229	1.4766
**Current pregnancy wanted**			0.339	1.0791			0.188	1.6755			0.793	0.23091
Later	8.26	11.22			32.07	23.76			21.68	12.39		
Then	84.72	78.61			42.09	60.81			65.52	72.70		
Not at all	7.01	10.17			25.84	15.43			12.80	14.91		

	Asia and Melanesia				Latin America and Caribbean[a]				North Africa/West Asia/Eastern Europe			
	Sought help (%)	Did not seek help (%)	*p-value*	chi-square or t-value	Sought help (%)	Did not seek help (%)	*p-value*	chi-square or t-value	Sought help (%)	Did not seek help (%)	*p-value*	chi-square or t-value
Partner/family characteristics												
**Partner's age (years)**			0.840	-0.2019			0.932	-0.085104			0.516	0.64933
<21	1.09	1.13			2.60	2.03			1.25	0.00		
21-30	23.80	27.30			29.02	29.35			20.88	25.57		
31-40	34.58	33.04			33.82	35.80			35.32	29.41		
41-50	29.03	25.55			24.01	20.18			27.41	34.16		
51-60	10.36	10.59			7.78	9.96			12.14	7.83		
>60	1.14	2.40			2.77	2.68			3.00	3.03		
**Partner's education**			**<0.001****	**7.3737**			0.425	0.847			0.919	0.082229
No education	37.71	46.12			9.61	12.48			11.05	11.95		
Primary	22.53	18.22			57.25	53.58			9.94	9.24		
Secondary or higher	39.76	35.66			33.14	33.94			79.01	78.82		
**Partner is working**			0.286	1.1372			0.938	0.0059832			0.457	0.5541
No	5.86	4.96			0.69	0.73			10.39	12.57		
Yes	94.14	95.04			99.31	99.27			89.61	87.43		
**Partner drinks alcohol**			0.070	2.6784			0.544	0.60612			0.742	0.41377
Never	1.88	2.85			11.16	12.39			2.54	4.65		
Often	45.09	38.27			47.54	42.41			56.93	55.40		
Sometimes	53.03	58.89			41.30	45.20			40.53	39.95		
**Number of living children (mean)**	3.54 (0.06)	3.67 (0.08)	0.199	-1.2851	2.95 (0.06)	3.06 (0.07)	0.238	-1.1795	2.39 (0.08)	2.43 (0.09)	0.713	-0.36774
**Women's father "beat" her mother**			0.677	0.17329			0.0811	3.0436			0.922	0.0096566
No	42.63	43.54			50.63	55.47			57.65	57.23		
Yes	57.37	56.46			49.37	44.53			42.35	42.77		
**Afraid of partner**			**<0.001****	**9.4015**			0.472	0.74705			0.229	1.4774
Never	9.78	8.40			35.46	38.10			21.55	20.47		
Most of the time	56.55	49.68			29.02	25.23			46.15	38.16		
Sometimes	33.68	41.92			35.52	36.67			32.30	41.37		
Community/societal characteristics												
**Place of residence**			0.761	0.092917			0.355	0.85562			0.684	0.16601
Urban	21.81	21.19			72.39	70.35			45.75	47.44		
Rural	78.19	78.81			27.61	29.65			54.25	52.56		
**Wealth index**			0.134	2.0174			0.546	0.58832			0.393	0.9352
Poorer or Poorest	49.99	49.29			43.15	41.00			45.29	48.71		
Middle	21.02	24.27			25.24	24.67			21.31	16.94		
Richer or Richest	29.00	26.44			31.60	34.33			33.40	34.35		
**Decision making**			**0.021***	**3.8672**			0.613	0.47752			**0.020***	**3.929**
Woman alone	16.66	12.60			42.02	41.10			35.57	24.50		
Woman and partner	38.77	44.17			49.11	48.17			48.02	51.00		
Partner alone	44.57	43.23			8.87	10.72			16.42	24.50		

	Asia and Melanesia				Latin America and Caribbean[a]				North Africa/West Asia/Eastern Europe			
	**Sought help (%)**	**Did not seek help (%)**	***p-value***	**chi-square or t-value**	**Sought help (%)**	**Did not seek help (%)**	***p-value***	**chi-square or t-value**	**Sought help (%)**	**Did not seek help (%)**	***p-value***	**chi-square or t-value**
**Controlling behavior: Partner...**												
jealous if respondent talks with other men/women	75.82	69.18	**<0.001****	**11.808**	73.96	66.46	**0.002***	**9.6439**	80.34	70.86	**0.007***	**7.3942**
accuses respondent of unfaithfulness	48.55	38.34	**<0.001****	**24.768**	58.67	48.56	**<0.001****	**14.262**	36.37	27.24	**0.020***	**5.4481**
does not permit respondent to meet female/male friends	41.27	34.13	**<0.001****	**11.713**	52.18	43.95	**0.003***	**8.9116**	37.83	36.62	0.770	0.085318
tries to limit respondent's contact with family	35.93	28.50	**<0.001****	**14.561**	38.03	29.54	**<0.001****	**12.087**	33.26	30.47	0.479	0.50254
insists on knowing where respondent is	59.46	46.28	**<0.001****	**39.471**	61.76	56.03	**0.033***	**4.5266**	70.15	61.14	**0.018***	**5.5782**
**Wife beating justified if wife**												
goes out without telling husband	52.21	56.88	**0.029***	**4.7529**	2.76	3.94	0.137	2.2177	24.90	32.80	**0.044***	**4.0694**
neglects the children	49.20	49.19	0.994	0.000061	4.79	6.61	0.112	2.5301	27.11	40.64	**<0.001****	**11.201**
argues with husband	52.35	53.31	0.653	0.20184	2.32	3.01	0.432	0.61653	15.25	27.26	**<0.001****	**11.66**
refuses to have sex with husband	37.05	32.76	**0.029***	**4.7899**	1.40	2.57	0.107	2.6055	14.12	25.01	**0.002***	**9.5733**
burns the food	28.53	25.92	0.156	2.013	2.44	3.75	0.162	1.961	7.70	16.53	**0.001***	**10.247**
**Health-seeking barriers (Following is a "big problem")**												
getting permission to go	42.45	44.98	0.107	2.2507	16.28	19.51	0.354	1.0826	14.08	19.02	0.135	2.2337
getting money needed for treatment	62.47	59.23	0.052	3.0133	70.70	67.95	0.338	1.0805	49.87	53.20	0.441	0.59314
distance to health care facility	57.98	56.97	0.863	0.1414	41.23	36.85	0.358	1.0269	32.63	33.80	0.579	0.50543
not wanting to go alone	52.91	52.70	0.385	0.94636	31.38	27.31	0.168	1.7924	31.98	34.40	0.558	0.34356

Notes. All variables are expressed as proportions (in %) except for age and number of children (mean and standard deviation). Age variables are presented in years. All results are weighted. Chi-square or t-value and p-value concern the help-seeking of IPV-exposed women during pregnancy. [a] For Peru, only unweighted data was available, hence Peru's data is excluded from regional weighted estimates.

***** p-value < 0.05. ****** p-value < 0.001.
