# Supplementary material for: Help-seeking behaviors among survivors of intimate partner violence during pregnancy in 54 low- and middle-income countries: evidence from Demographic and Health Survey data
Source: BMC Public Health. 2025 Feb 1;25:413. doi: 10.1186/s12889-025-21421-3 (PMC11787739; doi:10.1186/s12889-025-21421-3)
Supplement: Supplementary file 2 — Additional file 2. Supplementary Material 2. [file 12889_2025_21421_MOESM2_ESM.pdf]

Supplement: Table 2. Descriptive statistics among women experiencing IPV during pregnancy, by region, weighted.

	Sub-Saharan Africa				Total[a]			
	Sought help (%)	Did not seek help (%)	*p-value*	chi-square or *t*-value	Sought help (%)	Did not seek help (%)	*p-value*	chi-square or *t*-value
Women's characteristics								
**Woman's age (years)**			**0.049***	**2.1168**			0.328	1.1552
15-19	3.95	6.30			3.26	3.89		
20-24	17.69	18.70			14.66	15.44		
25-29	22.62	22.36			19.50	20.03		
30-34	19.82	19.20			19.40	17.79		
35-39	15.08	14.63			17.19	16.28		
40-44	11.41	10.36			13.15	13.27		
45-49	9.43	8.45			12.84	13.29		
**Age at first cohabitation (years)**			0.311	1.0132			**0.011***	**2.5507**
<15	13.42	13.50			12.85	15.27		
15-19	56.32	57.19			55.71	54.02		
20-24	22.88	22.56			24.12	24.22		
25+	7.38	6.75			7.32	6.48		
**Marital status**			**<0.001****	**18.224**			**<0.001****	**55.814**
Previously married	25.87	19.38			25.34	16.85		
Currently married	74.13	80.62			74.66	83.15		
**Woman's education**			**<0.001****	**10.042**			**<0.001****	**62.664**
No education	27.10	33.94			29.46	42.09		
Primary	48.11	42.67			34.68	27.54		
Secondary or higher	24.79	23.40			35.86	30.37		
**Woman is working**			**0.002***	**10.065**			**<0.001****	**91.215**
No	23.27	27.85			41.27	52.40		
Yes	76.73	72.15			58.73	47.60		
**Who earns more**			0.928	0.073918			0.686	0.37611
About the same	10.99	11.41			14.70	14.95		
Husband/partner earns more	77.89	77.07			71.51	72.37		
Woman earns more than him	11.12	11.53			13.78	12.68		
**Exposure to mass media (at least once a week)**								
Reading newspaper or magazine	7.38	5.92	**0.008***	**4.8833**	11.86	10.01	**<0.001****	**14.118**
Listening to radio	36.97	36.47	0.055	2.9039	33.31	29.80	**<0.001****	**7.3545**
Watching television	24.76	26.45	0.405	0.90348	40.88	42.88	0.203	1.5979
**Current pregnancy wanted**			0.548	0.59669			0.347	1.059
Later	32.39	35.32			23.31	19.85		
Then	56.58	56.23			65.66	70.01		
Not at all	11.03	8.46			11.03	10.14		

	Sub-Saharan Africa				Total[a]			
	Sought help (%)	Did not seek help (%)	*p-value*	chi-square or *t*-value	Sought help (%)	Did not seek help (%)	*p-value*	chi-square or *t*-value
Partner/family characteristics								
**Partner's age (years)**			0.293	-1.0515			0.369	-0.89831
<21	0.75	1.13			1.06	1.14		
21-30	25.24	26.74			24.70	27.20		
31-40	38.56	35.48			36.48	33.76		
41-50	24.53	23.66			26.34	25.05		
51-60	7.94	10.00			9.12	10.22		
>60	2.98	2.99			2.30	2.63		
**Partner's education**			**0.004***	**5.5096**			**<0.001****	**38.168**
No education	22.06	26.06			25.25	35.08		
Primary	40.69	35.34			33.65	26.19		
Secondary or higher	37.25	38.60			41.10	38.74		
**Partner is working**			0.233	1.4219			0.416	0.66119
No	4.22	3.44			4.92	4.51		
Yes	95.78	96.56			95.08	95.49		
**Partner drinks alcohol**			**0.001***	**6.663**			**<0.001****	**10.37**
Never	6.95	10.51			5.89	7.35		
Often	52.21	45.38			50.25	42.56		
Sometimes	40.84	44.11			43.86	50.09		
**Number of living children (mean)**	3.61 (0.04)	3.57 (0.06)	0.551	0.5963	3.37 (0.03)	3.46 (0.04)	0.101	-1.6416
**Women's father "beat" her mother**			0.116	2.4717			0.930	0.0076428
No	53.37	56.09			50.06	50.16		
Yes	46.63	43.91			49.94	49.84		
**Afraid of partner**			**<0.001****	**23.788**			**<0.001****	**7.2668**
Never	21.02	29.88			18.74	18.59		
Most of the time	40.93	31.54			45.21	41.10		
Sometimes	38.05	38.58			36.05	40.31		
Community/societal characteristics								
**Place of residence**			0.735	0.11469			**0.007***	**7.1874**
Urban	33.22	33.78			38.86	35.64		
Rural	66.78	66.22			61.14	64.36		
**Wealth index**			0.232	1.4636			0.072	2.649
Poorer or Poorest	47.24	46.04			47.05	46.73		
Middle	19.57	21.88			21.25	23.32		
Richer or Richest	33.20	32.08			31.70	29.96		
**Decision making**			0.068	2.6882			**0.004***	**5.6412**
Woman alone	17.31	17.05			22.30	18.77		
Woman and partner	42.72	38.94			42.88	43.55		
Partner alone	39.98	44.01			34.82	37.67		

	Sub-Saharan Africa				Total[a]			
	Sought help (%)	Did not seek help (%)	*p-value*	chi-square or *t*-value	Sought help (%)	Did not seek help (%)	*p-value*	chi-square or *t*-value
**Controlling behavior: Partner...**								
jealous if respondent talks with other men/women	78.51	71.50	**<0.001****	**25.236**	76.94	69.38	**<0.001****	**51.989**
accuses respondent of unfaithfulness	53.16	45.09	**<0.001****	**23.516**	51.88	41.73	**<0.001****	**76.356**
does not permit respondent to meet female/male friends	45.57	38.73	**<0.001****	**17.44**	45.21	37.49	**<0.001****	**43.057**
tries to limit respondent's contact with family	36.13	29.53	**<0.001****	**17.266**	36.27	29.09	**<0.001****	**43.539**
insists on knowing where respondent is	68.17	59.96	**<0.001****	**27.829**	64.55	52.84	**<0.001****	**102.34**
**Wife beating justified if wife**								
goes out without telling husband	35.35	39.46	**0.009***	**6.7607**	32.59	40.22	**<0.001****	**47.128**
neglects the children	40.48	43.78	**0.043***	**4.0845**	34.66	38.71	**<0.001****	**13.17**
argues with husband	34.98	38.62	**0.022***	**5.2322**	31.66	37.77	**<0.001****	**30.252**
refuses to have sex with husband	30.17	35.65	**<0.001****	**12.579**	25.07	27.09	**0.042***	**4.1476**
burns the food	19.96	22.89	**0.031***	**4.6688**	17.84	20.10	**0.011***	**6.3902**
**Health-seeking barriers (Following is a "big problem")**								
getting permission to go	19.51	23.35	**0.006***	**7.6383**	26.23	34.28	**<0.001****	**49.68**
getting money needed for treatment	64.16	65.06	0.581	0.30483	63.26	61.63	**0.007***	**5.0392**
distance to health care facility	42.56	43.57	0.542	0.3727	46.72	49.52	**<0.001****	**12.026**
not wanting to go alone	23.87	24.97	0.443	0.58744	34.61	40.50	**<0.001****	**32.91**

Notes. All variables are expressed as proportions (in %) except for age and number of children (mean and standard deviation). Age variables are presented in years. All results are weighted. Chi-square or t-value and p-value concern the help-seeking of IPV-exposed women during pregnancy. [a] For Peru, only unweighted data was available, hence Peru's data is excluded from total weighted estimates. ***** p-value < 0.05. ****** p-value < 0.001.
